# Supplementary material for: A standalone bismuth vanadate-silicon artificial leaf achieving 8.4% efficiency for hydrogen production
Source: Nat Commun. 2025 Mar 21;16:2792. doi: 10.1038/s41467-025-58102-z (PMC11928484; doi:10.1038/s41467-025-58102-z)
Supplement: Supplementary file 1 — Supplementary Information [file 41467_2025_58102_MOESM1_ESM.pdf]

Supplementary Information

A standalone bismuth vanadate-silicon artificial leaf achieving 8.4% efficiency for hydrogen production

Boyan Liu[1], Xin Wang[1], Yingjuan Zhang[1], Mingshan Zhu[2]*, Chenxin Zhang[3], Shaobin Li[3], Yanhang Ma[4], Wei Huang[1]* & Songcan Wang[1,5]*

[1] State Key Laboratory of Flexible Electronics & Institute of Flexible Electronics, Northwestern Polytechnical University, 127 West Youyi Road, Xi'an, 710072, China.

[2] Guangdong Key Laboratory of Environmental Pollution and Health, School of Environment, Jinan University, Guangzhou, 511443, China.

[3] College of the Environment and Ecology, Xiamen University, Xiamen 361102, China.

[4] School of Physical Science and Technology & Shanghai Key Laboratory of High-resolution Electron Microscopy, ShanghaiTech University, Shanghai 201210, China.

[5] Research & Development Institute of Northwestern Polytechnical University in Shenzhen, Sanhang Science & Technology Building, No. 45th, Gaoxin South 9th Road, Nanshan District, Shenzhen 518063, China.

*Corresponding authors E-mail: zhumingshan@jnu.edu.cn, iamwhuang@nwpu.edu.cn, iamscwang@nwpu.edu.cn

Supplementary Methods

All applied potentials versus a Ag/AgCl electrode were converted to the potentials versus the reversible hydrogen electrode (RHE) using the Nernst equation below:[1]

$$E_{\mathrm{RHE}} = E_{\mathrm{Ag/AgCl}} + E_{\mathrm{Ag/AgCl}}(\mathrm{ref}) + 0.0591\mathrm{V} \times \mathrm{pH} \quad (1)$$

$$E_{\mathrm{Ag/AgCl}}(\mathrm{ref}) = 0.1976 \text{ V vs. NHE at 25 °C}$$

where E_{RHE} refers to the converted potential versus RHE. The value of $E_{\mathrm{Ag/AgCl}}(\mathrm{ref})$ is 0.1976 V at ambient temperature (25 °C) and $E_{\mathrm{Ag/AgCl}}$ is the obtained potential versus Ag/AgCl.

Applied bias photon-to-current efficiency (ABPE) can be calculated using the following equation:[2]

$$\mathrm{ABPE} = \left[\frac{J_{\mathrm{ph}}(\mathrm{mA{\bullet}cm^{-2}}) \times (1.23 - V_{bias})(\mathrm{V})}{P_{\mathrm{total}}(\mathrm{mW{\bullet}cm^{-2}})} \right]_{\mathrm{AM1.5G}} \quad (2)$$

Where J_{ph} is the photocurrent density obtained under an applied bias (V_{bias}), and P_{total} is the incident illumination power density.

Incident-photon-to-current conversion efficiency (IPCE) was obtained using an Oriel Cornerstone 260 1/4 m monochromator coupled with a 300 W Oriel Xe lamp as the simulated light source. An applied potential of 1.23 V vs. RHE was supplied by a CHI 760E electrochemical workstation and the power density at a specific wavelength was measured by a Newport 1918-c power meter. IPCE values were calculated using Equation 3.[2]

$$\mathrm{IPCE}(\%)=\frac{J_{\mathrm{ph}}(\mathrm{mA{\bullet}cm^{-2}})\times 1239.8(\mathrm{V}\times\mathrm{nm})}{\lambda(\mathrm{nm})\times P_{\mathrm{light}}(\mathrm{mW{\bullet}cm^{-2}})}\times 100\% \tag{3}$$

where 1239.8 (V nm) represents a multiplication of *h* (Planck's constant) and *c* (the speed of light), λ is the incident light wavelength (nm), and P_{light} is the monochromatic illumination power intensity.

Electrochemical impedance spectroscopy (EIS) spectra were collected with an AC voltage amplitude of 10 mV at the open circuit potentials of the films under AM 1.5G illumination (frequency range: 0.01 Hz~100 kHz).

The Mott-Schottky (MS) spectra were measured at a voltage window of 0~0.6 V vs. RHE in the dark (increment: 10 mV, frequency: 1 kHz). According to the MS curves, the charge carrier density (N_d) can be calculated using the following equation:[3]

$$\frac{1}{C^2}=\frac{2}{\varepsilon\varepsilon_0 eN_{\mathrm{d}}}\times\left(V-V_{\mathrm{fb}}-\frac{k_{\mathrm{B}}T}{q}\right) \tag{4}$$

$$N_{\mathrm{d}}=\frac{2}{e\varepsilon\varepsilon_0}\times\left(\frac{\mathrm{d}\left(\frac{1}{C^2}\right)}{\mathrm{d}V_{\mathrm{S}}}\right)^{-1} \tag{5}$$

where C is the space-charge capacitance, V (V vs. RHE) is the applied voltage, V_{fb} (V vs. RHE) is the flat-band potential, N_d is the charge carrier density, ε is the dielectric constant of the semiconductor (taken as 68 for the $BiVO_4$[4]), ε_0 is the vacuum permittivity (8.854 × 10^{-12} F^{-1} m^{-1}), k_{B} is Boltzmann's constant (1.381 × 10^{-23}J K^{-1}), e is the electronic charge (1.602 × 10^{-19} C), and T is the absolute temperature.[5]

$$J_{\mathrm{ph}}=J_{\mathrm{abs}}\times\eta_{\mathrm{sep}}\times\eta_{\mathrm{trans}} \tag{6}$$

$$J_{\mathrm{abs}} = \frac{q}{hc}\int_{300}^{520} \lambda \phi_{\lambda} \eta_{\mathrm{LHE}} \mathrm{d}\lambda \quad (7)$$

$$\eta_{\mathrm{sep}} = J^{\mathrm{Na_2SO_3}} / J_{\mathrm{abs}} \quad (8)$$

$$\eta_{\mathrm{trans}} = J^{\mathrm{KBi}} / J^{\mathrm{Na_2SO_3}} \quad (9)$$

$$\eta_{\mathrm{LH}} = 1 - 10^{-A(\lambda)} \quad (10)$$

The J_{abs} is the photon adsorption rate expressed as the photocurrent density, η_{sep} is the charge separation efficiency in the bulk of a photoanode, η_{trans} is the surface charge transfer efficiency of a photoanode, *q* is the charge of an electron, *h* is the Plank constant, *c* is the light speed, ϕ_{λ} is the photon flux of the AM 1.5G solar spectrum, and η_{LH} is the light absorption efficiency of a monochromatic light. The $J^{\mathrm{Na_2SO_3}}$ is the photocurrent density measured in 1 M potassium borate buffer with 0.2 M Na_2SO_3 electrolyte (pH=9.5), which serves as a hole scavenger and ensures the hole injection rate approaching 100%, and J^{KBi} is the photocurrent density measured in 1 M potassium borate buffer (pH=9.5). The light absorbance (*A*) is measured experimentally by UV-Vis spectroscopy, λ is wavelength.[6]

The estimated photocurrent densities (J_c) were calculated by integrating the IPCE values with the standard solar spectrum (ASTMG-173-03) using the following equation: [7]

$$J_{\mathrm{c}} = \int_{300}^{600} \frac{\lambda \times \mathrm{IPCE}(\lambda) \times E(\lambda)}{1240} d\lambda \quad (11)$$

Specifically, λ and $E(\lambda)$ represent the light wavelength (nm) and the corresponding power density (mW cm^{-2}), respectively.

Biexponential function fitting was employed to analyze the time-resolved photoluminescence decay curves, and the average recombination lifetime (τ_{avg}) was calculated by the following Equations:[8]

$$R(t) = A_1 e^{-\frac{t}{\tau_1}} + A_2 e^{-\frac{t}{\tau_2}} \tag{12}$$

$$\tau_{avg} = \frac{A_1\tau_1^2 + A_2\tau_2^2}{A_1\tau_1 + A_2\tau_2} \tag{13}$$

In the formula, τ_1 and τ_2 are defined as the decay time for the fluorescence intensity, representing the speed of carrier recombination.

The empirical expression relating the V-O bond length to the Raman stretching frequencies is as follow:[9]

$$v(\mathrm{cm^{-1}}) = 21349 \times e^{-1.9176R(\text{Å})} \tag{14}$$

Where v is the Raman shift ($\mathrm{cm^{-1}}$) and R is the V-O bond length (Å).

The Faraday efficiency calculation equations is as follows：

$$\eta_{\mathrm{F}}(\%) = \frac{\text{moles of gas product actually measured}}{\text{theoretical moles based on the measured current}} \times 100 \tag{15}$$

$$\mathrm{FE_{H2}}(\%) = \frac{n_{\mathrm{H_2}}}{\frac{Q}{2F}} \times 100 = \frac{\mathrm{moles_{H_2}} \times 2 \times 96,485}{Q} \times 100 \tag{16}$$

$$\mathrm{FE_{O_2}}(\%) = \frac{n_{\mathrm{O_2}}}{\frac{Q}{4F}} \times 100 = \frac{\mathrm{moles_{O_2}} \times 4 \times 96,485}{Q} \times 100 \tag{17}$$

where $n_{\mathrm{H_2}}$ and $n_{\mathrm{O_2}}$ are the moles of H_2 and O_2, respectively, quantified by GC during the stability test in the two- or three-electrode set-up, the Faraday constant (F)

is 96,485 C mol^{-1} and Q/2 (e^{-}/2) and Q/4 (e^{-}/4) are the charges passed in Coulombs (C) for H_2 and O_2, respectively.

The solar-to-hydrogen (STH) efficiency of the two-electrode setup was calculated using the following equation：

$$\mathrm{STH}(\%)=\frac{J_{\mathrm{op}}\times 1.23\ \mathrm{V}}{P_{\mathrm{in}}}\times 100 \tag{18}$$

where J_{op} is the operating photocurrent density (mA cm^{-2}) obtained during chronoamperometry (J–t) measurements at "zero" applied potential in the two-electrode set-up, P_{in} is the solar incident light power density (100 mW cm^{-2}) and 1.23 V is the thermodynamic potential for overall water splitting.

Based on the gas production rate measurements, we collected data on H_2 release and calculated the STH efficiency using the following equation:

$$\mathrm{STH}(\%)=\left[\frac{n_{\mathrm{H_2}}\left(\mathrm{mmol\ H_2{\bullet}s^{-1}}\right)\times \Delta G\left(237{,}000\ \mathrm{J{\bullet}mol^{-1}}\right)}{P_{\mathrm{in}}(\mathrm{mW{\bullet}cm^{-2}})\times A(\mathrm{cm^{2}})}\right]_{\mathrm{AM\ 1.5}} \tag{19}$$

where $n_{\mathrm{H_2}}$ is the mmol of H_2 gas per second measured by GC, A is the illumination area of the PEC-PV devices, P_{in} is the solar incident light power density (100 mW cm^{-2}) and ΔG is Gibb's free energy (237,000 J mol^{-1}) for overall water splitting.

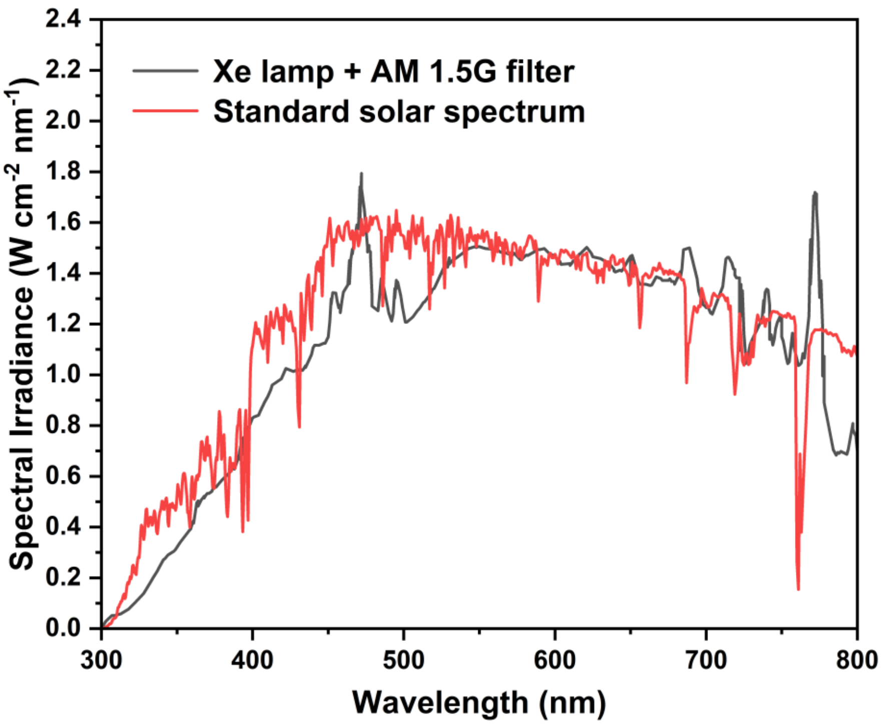

Supplementary Fig. 1. Spectrum of the Xe lamp light equipped with an AM 1.5G filter. Spectral irradiance of the Xe lamp light equipped with an AM 1.5G filter in the range of 300-800 nm was carefully calibrated to well match that of the standard AM 1.5G spectrum. Source data are provided as a Source Data file.

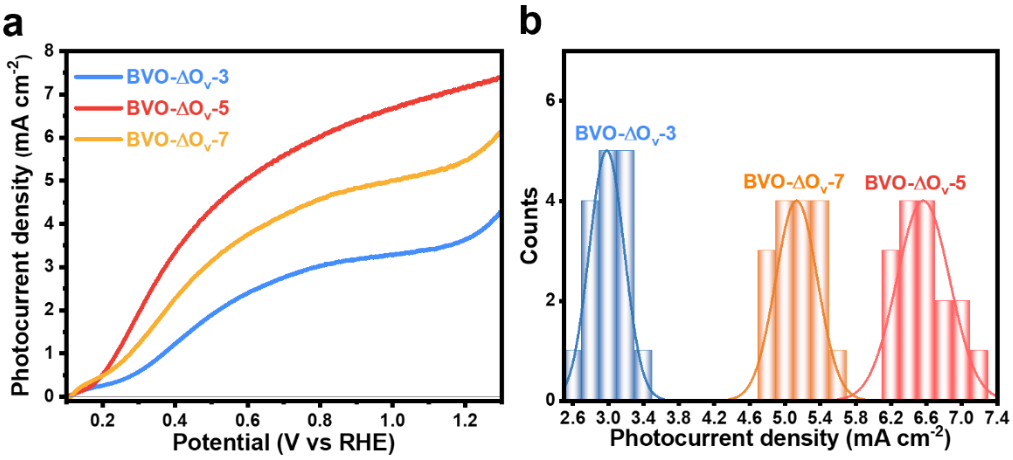

Supplementary Fig. 2. PEC performances of the BVO-ΔO$_v$-3, BVO-ΔO$_v$-5 and BVO-ΔO$_v$-7 photoanodes. a Photocurrent density versus potential curves, and (**b**) photocurrent density distribution in a 1 M borate buffer electrolyte with 0.2 M Na_2SO_3 (pH 9.5) under AM 1.5G illumination. Source data are provided as a Source Data file.

Supplementary Discussion

When the electrodeposition time is 1 min, the BSO-5-1min film shows a similar sponge-like structure, but the coverage is inhomogeneous (Supplementary Fig. 3a). When the electrodeposition time is increased to 3 min, the sponge-like structure and some hexagon particles can be observed in the BSO-5-3min film (Supplementary Fig. 3b). When the electrodeposition time is increased to 4 min, more obvious hexagon particles can be observed (Supplementary Fig. 3c). For the converted BVO-ΔO_v-1min film, the coverage is low and the thickness is only around 650 nm (Supplementary Fig. 3d). However, longer electrodeposition time leads to a significantly higher film thickness (insets in Supplementary Figs. 3e, f).

As shown in Supplementary Fig. 4, the PEC sulfide oxidation performances of these samples are lower than that of the BVO-ΔO_v-5 sample (Supplementary Fig. 2a). Since $VO(C_5H_7O_2)_2$ is commonly applied as the vanadium source for the conversion of bismuth precursor films to $BiVO_4$ films[10-12], such a high photocurrent density should be attributed to the BSO precursor films obtained by our electrolyte recipe.

When the nitric acid is replaced by acetic acid with the same pH value, the bismuth precursor film obtained under the same electrodeposition conditions exhibits a relatively dense morphology with micropores (Supplementary Fig. 5a), which is different from the sponge-like structure shown in Fig. 1b. XRD pattern reveals that the precursor film is composed of a mixture of $Bi_{34.7}O_{36}(SO_4)_{16}$ and metallic Bi (Supplementary Fig. 5b). Interestingly, the converted $BiVO_4$ film (denoted as BVO) exhibits a similar wormlike structure and film thickness (Supplementary Fig. 5c). The

histogram of 16 pieces of the BVO samples demonstrates the average photocurrent density of 4.2 mA cm^{-2} with a champion value of 4.5 mA cm^{-2} (Supplementary Fig. 5d), which is much lower than its BVO-ΔO_v-5 counterpart.

To investigate the effect of thiourea in the precursor electrolyte, another $BiVO_4$ sample was converted by a BiOI nanosheet array film according to a previous report[13]. By carefully tuning the electrodeposition time, the converted $BiVO_4$ film with a similar film thickness of approximately 1.3 μm was obtained (denoted as BVO-Ref, Supplementary Figs. 6a-c). Interestingly, the photocurrent density of the BVO-Ref is only 3.6 mA cm^{-2} at 1.23 V vs. RHE under AM 1.5G illumination (Supplementary Fig. 6d).

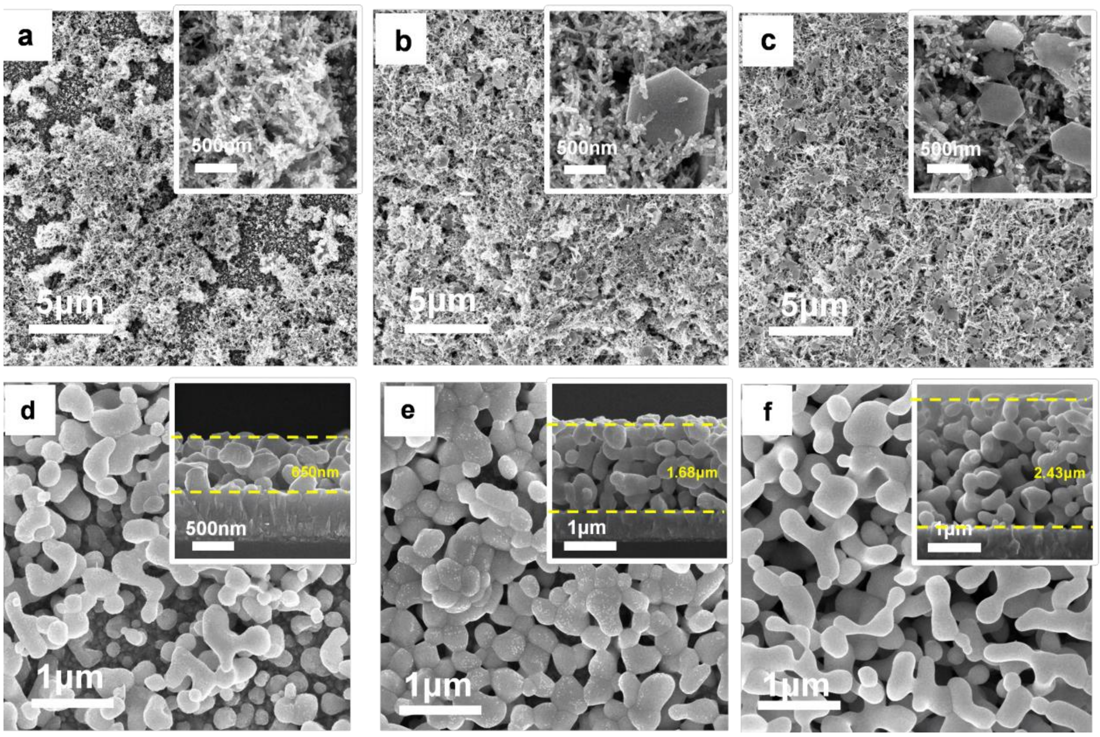

Supplementary Fig. 3. SEM images of the samples. a BSO-5-1min, (**b**) BSO-5-3min, and (**c**) BSO-5-4min. Insets: the large magnification views of the BSO films. **d** BVO-ΔO_v-1min, (**e**) BVO-ΔO_v-3min, and (**f**) BVO-ΔO_v-4min. Insets: the cross-sectional SEM images of the BVO-ΔO_v-*x*min films.

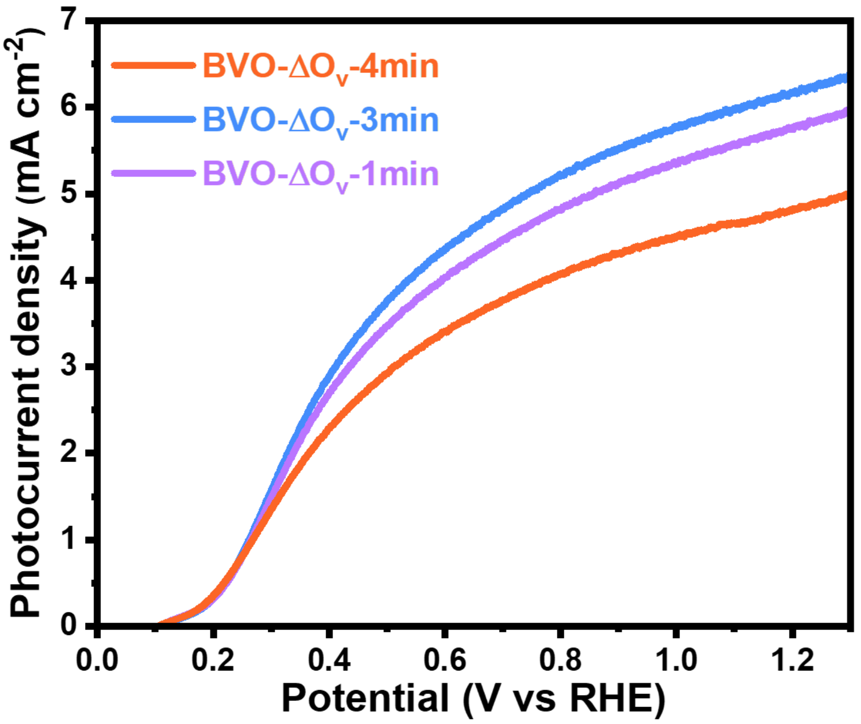

Supplementary Fig. 4. PEC performance of the BVO-ΔO$_v$-*x*min films. Photocurrent density versus potential curves of BVO-ΔO$_v$-1min, BVO-ΔO$_v$-3min and BVO-ΔO$_v$-4min in a 1 M borate buffer electrolyte with 0.2 M Na_2SO_3 (pH 9.5) under AM 1.5G illumination. Source data are provided as a Source Data file.

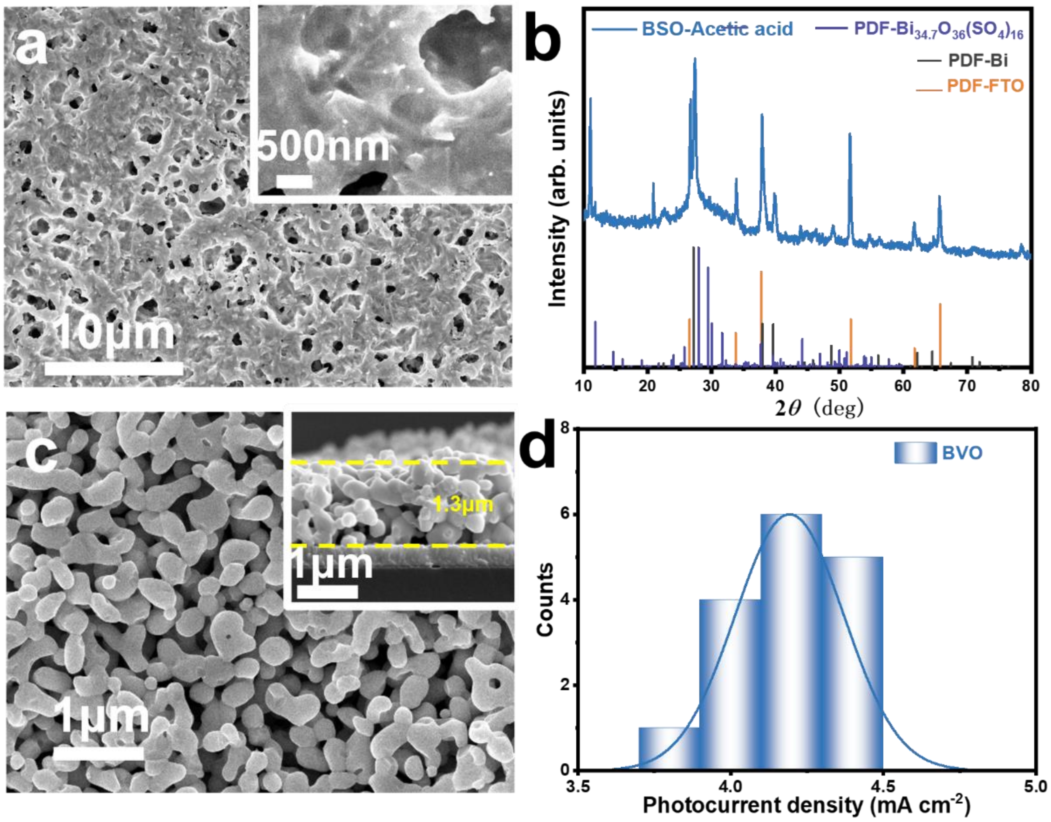

Supplementary Fig. 5. Material characterization and PEC performance of the BVO films. **a** SEM image of BSO-5Ace. Inset: the large magnification view of BSO-5Ace. **b** XRD pattern of BSO-5Ace. **c** SEM images of BVO. Inset: the cross-sectional SEM images of BVO. **d** Photocurrent density distribution of BVO in a 1 M borate buffer electrolyte with 0.2 M Na_2SO_3 (pH 9.5) under AM 1.5G illumination. Source data are provided as a Source Data file.

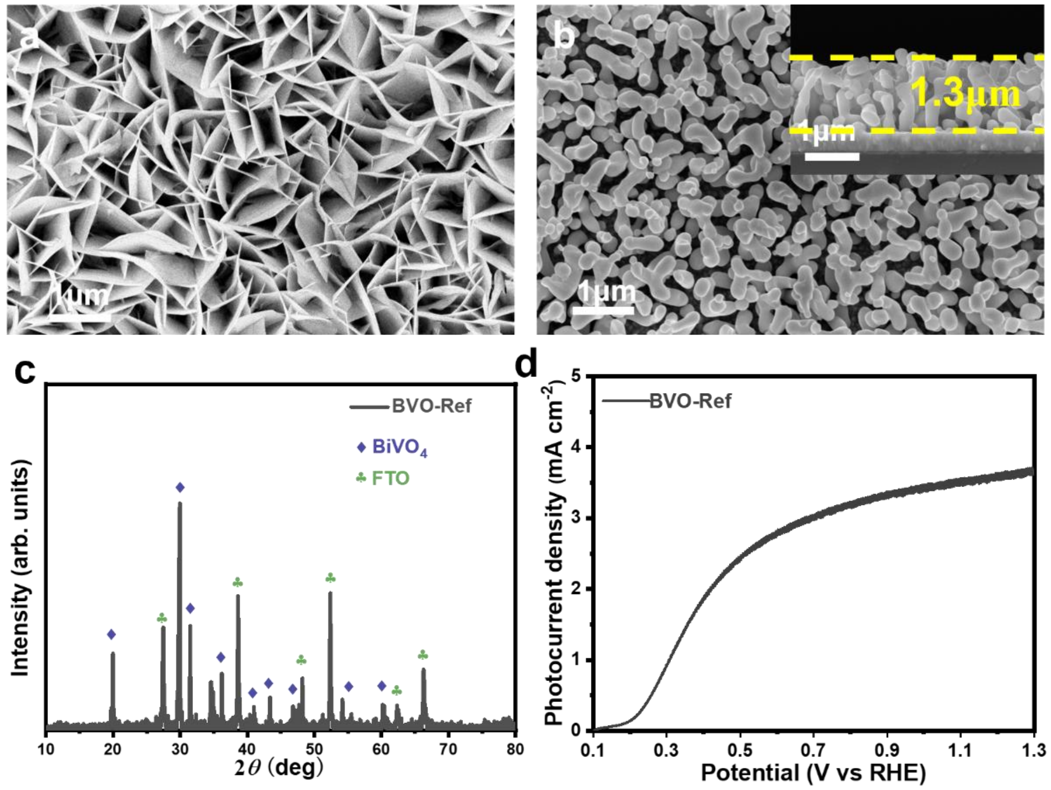

Supplementary Fig. 6. Material characterization and PEC performance of the BVO-Ref films. **a** SEM image of BiOI. **b** SEM image of BVO-Ref. Inset: the cross-sectional SEM images of BVO-Ref. **c** XRD pattern of BVO-Ref. **d** Photocurrent density versus potential curves of BVO-Ref in a 1 M borate buffer electrolyte with 0.2 M Na_2SO_3 (pH 9.5) under AM 1.5G illumination. Source data are provided as a Source Data file.

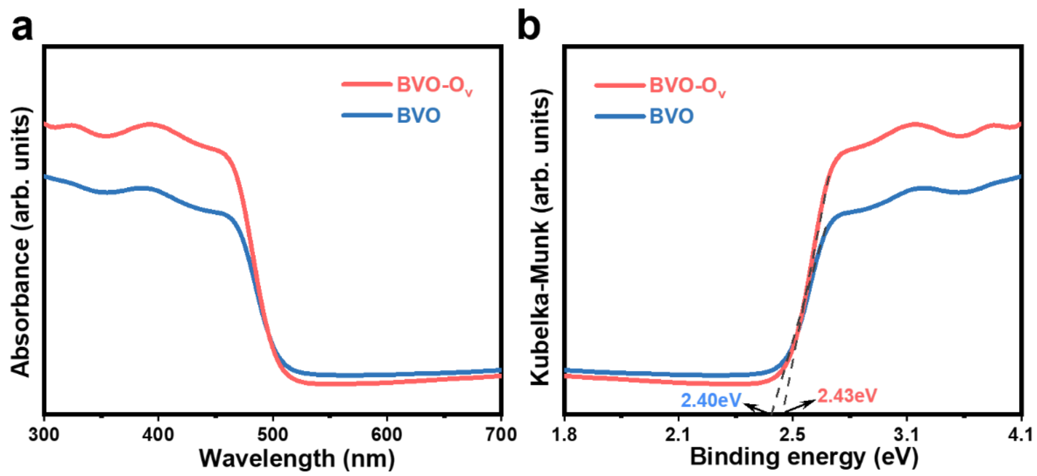

Supplementary Fig. 7. Light absorption properties of BVO-ΔO$_v$ and BVO. a UV-vis spectra. **b** Kubelka-Munk plots. Source data are provided as a Source Data file.

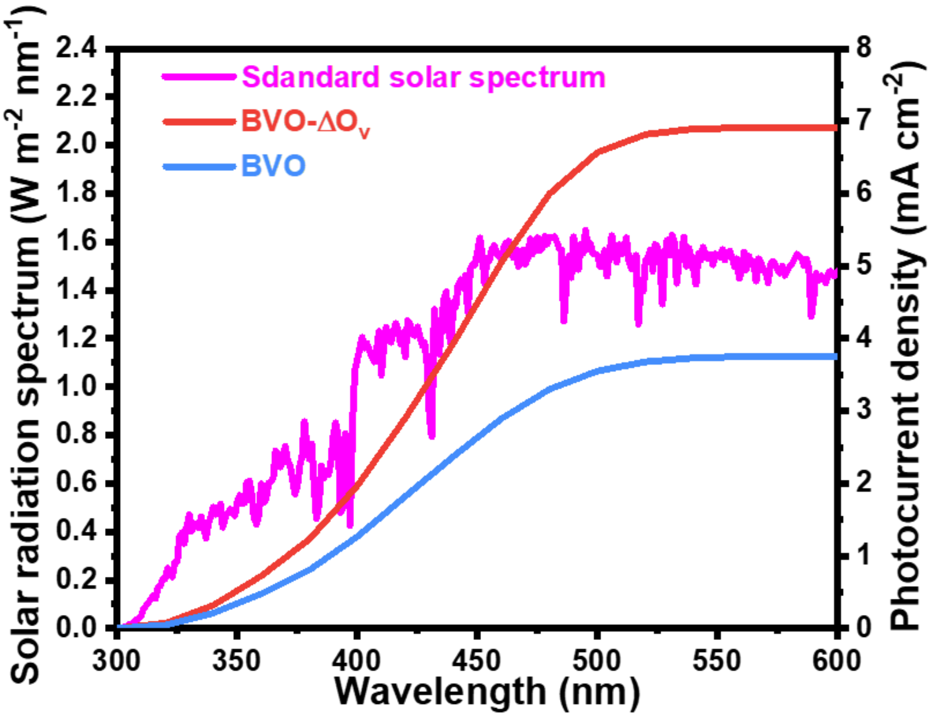

Supplementary Fig. 8. Calculated photocurrent density curves of the samples. Photocurrent density curves of BVO-ΔO$_v$ and BVO obtained by integrating their IPCE curves with standard AM 1.5G spectrum. Source data are provided as a Source Data file.

Supplementary Discussion

In the absence of Na_2SO_3, the photocurrent densities of both BVO-ΔO_v and BVO are much lower due to the sluggish OER kinetics on the $BiVO_4$ surfaces. As shown in Supplementary Fig. 9a, the photocurrent densities of BVO-ΔO_v and BVO are 4.15 and 3.0 mA cm^{-2} at 1.23 V vs. RHE under AM 1.5G illumination, respectively. BVO-ΔO_v exhibits an IPCE value of 62% in the wavelength range of 350-450 nm, while that of its BVO counterpart is 47% (Supplementary Fig. 9b).

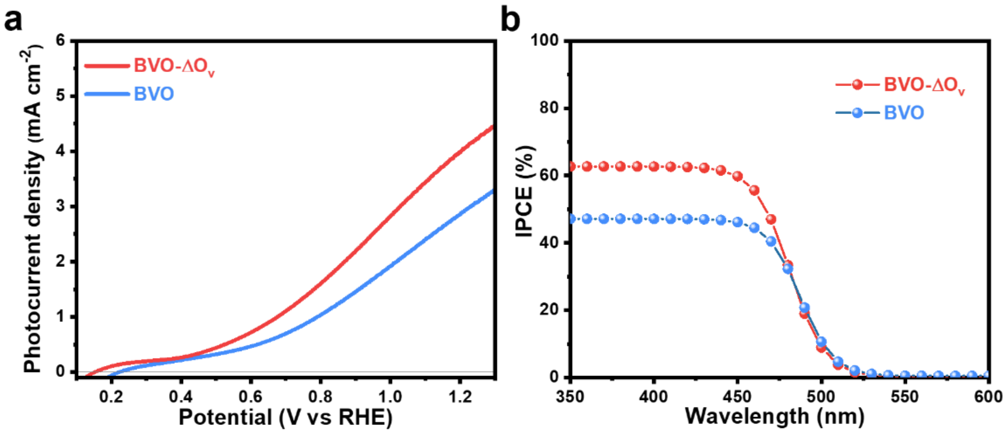

Supplementary Fig. 9. Photoelectrochemical performance of the samples in the absence of Na_2SO_3. a Photocurrent density versus potential curves and (**b**) IPCE curves at 1.23 V vs. RHE of BVO-ΔO_v and BVO in a 1 M borate buffer electrolyte (pH 9.5) under AM 1.5G illumination. Source data are provided as a Source Data file.

Supplementary Discussion

In order to study the interfacial kinetics of the BVO-ΔO_v and BVO films, their electrochemical impedance spectroscopy (EIS) curves were measured. As shown in Supplementary Fig. 10, both films exhibit a typical EIS curve consisting of only one semicircle, which can be fitted by an equivalent circuit model (inset in Supplementary Fig. 10) composed of a series resistance (R_s), a charge transfer resistance (R_{ct}) and a constant phase angle element (CPE)[14]. It is obvious that the charge transfer resistance of BVO-ΔO_v is significantly lower than that of its BVO counterpart (Supplementary Table 2), indicating the better surface charge transfer properties that alleviate the energy loss during PEC water splitting[15].

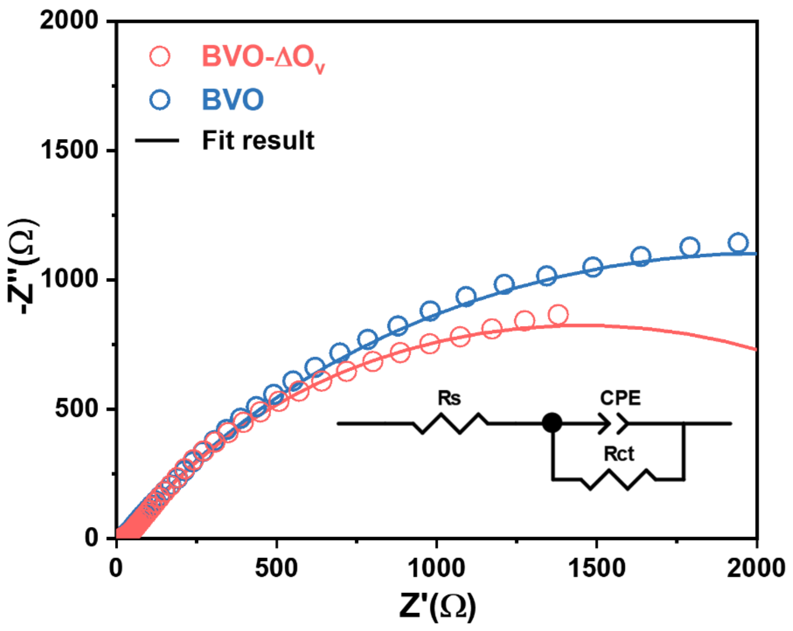

Supplementary Fig. 10. Interfacial charge transfer properties of BVO-ΔO_v and BVO. EIS curves of the BVO-ΔO_v and BVO films. Source data are provided as a Source Data file.

Supplementary Discussion

The symmetrical stretching modes of the V–O bond in the Raman spectrum (Supplementary Fig. 11) of BVO-ΔO$_V$ is 824.66 cm^{-1}, which is slightly shifted to a higher wavenumber compared to that of its BVO counterpart (821.31 cm^{-1}). According to Supplementary Equation (14), the V–O bond lengths in the BVO-ΔO$_V$ and BVO photoanodes are 1.697 and 1.699 Å, respectively. The shorter of the V–O bond length suggests the formation of more oxygen vacancies[16].

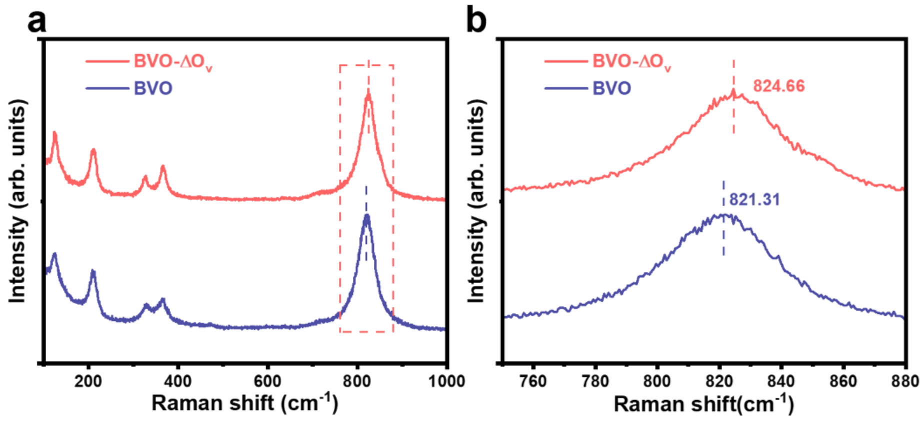

Supplementary Fig. 11. V–O bond length characterizations. **a** Raman spectra of BVO-ΔO$_V$ and BVO films. **b** Partial enlargement of the selective area in (**a**). Source data are provided as a Source Data file.

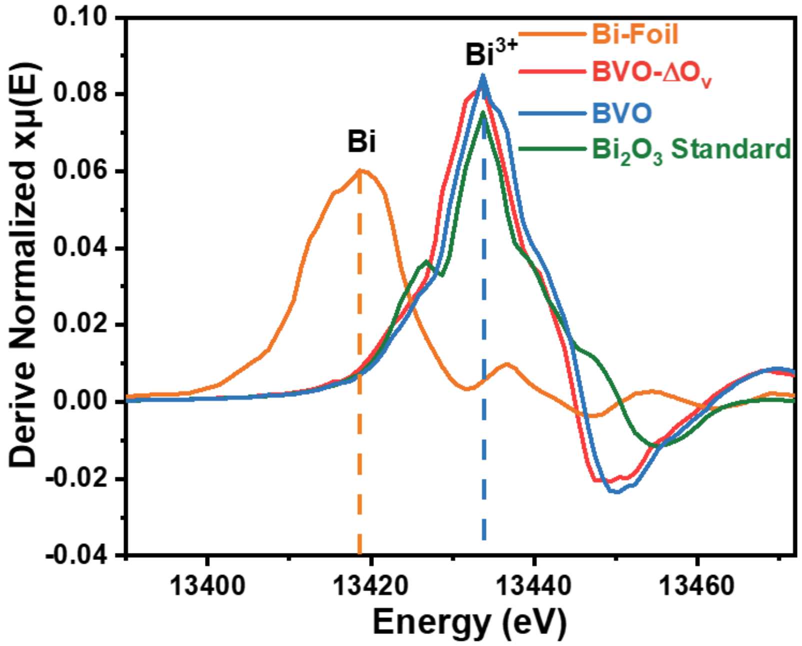

Supplementary Fig. 12. Derive normalized Bi *L*-edge XANES xμ(*E*) spectra. Comparison amongst the BVO-ΔO_v, BVO, Bi_2O_3 standard and Bi foil. Source data are provided as a Source Data file.

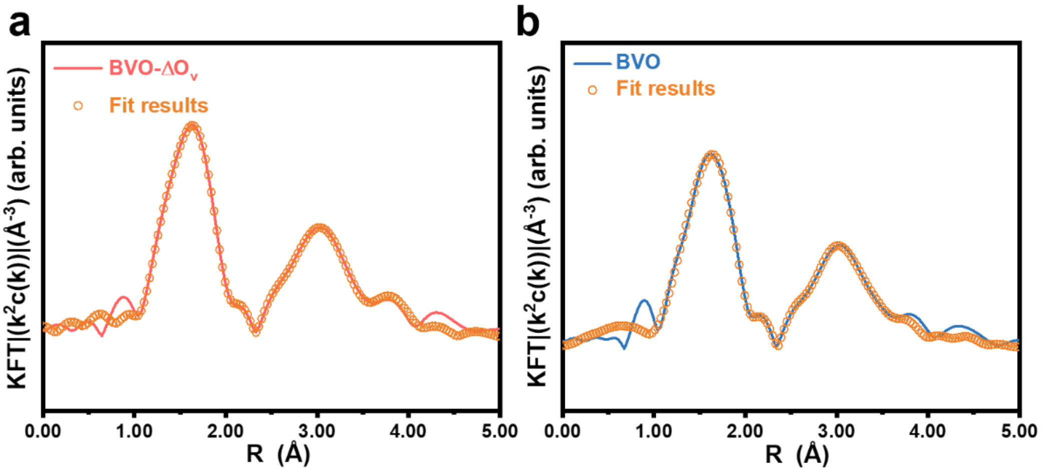

Supplementary Fig. 13. Fourier-transformed (FT)-Extended X-ray absorption fine structure (EXAFS) fitting curves at *R* space of Bi *L*-edge. a BVO-ΔO_v and (**b**) BVO. Source data are provided as a Source Data file.

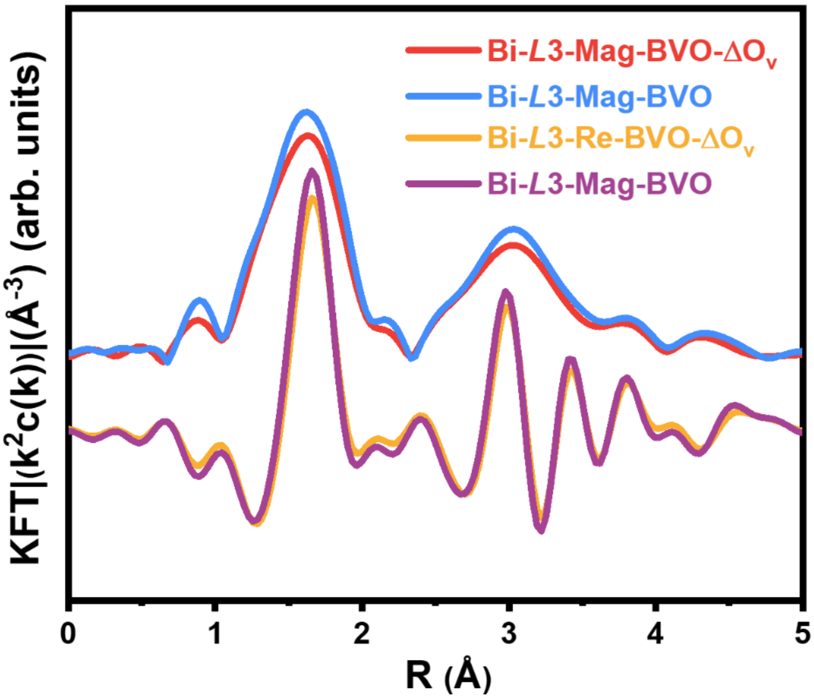

Supplementary Fig. 14. Magnitude and real part curves of the samples. Magnitude and real parts of BVO-ΔO_v and BVO. Source data are provided as a Source Data file.

Supplementary Discussion

Supplementary Fig. 15a shows the XANES spectra at V *K*-edge of BVO-ΔO_v, BVO, V foil, V_2O_3, and V_2O_5. An intense pre-edge peak located at 5469 eV can be observed in all samples, which is associated with 1*s*−3*d* transitions. The edge positions of both BVO-ΔO_v and BVO are almost overlapped, which is slightly negative shifted compared to that of V_2O_5, indicating the presence of low-valent V ions along with the V^{5+} ions in the samples. V *K*-edge radial distance $\chi(R)$ space spectra of BVO-ΔO_v and BVO were performed to obtained more information about the local structure around V ions (Supplementary Figs. 15b-d). As shown in Supplementary Fig. 15b, the peaks located at around 1.32 Å assigned to the V−O bond are observed in both BVO-ΔO_v and BVO samples, and no obvious peak shift can be observed. However, the slightly weaker intensity of the V−O peak in BVO-ΔO_v indicates the generation of more oxygen vacancies that leads to less amount of V−O bonds. The good fitting results of $\chi(R)$ and $\chi(k)$ space spectra (Supplementary Figs. 15c, d) with reasonable *R*-factors and the obtained fitting parameters (Supplementary Table 7) provide a quantitative of the V–O1 and V–O2 bonds. The shorter distance of the V–O2 bond in BVO-ΔO_v suggests the presence of more oxygen vacancies.

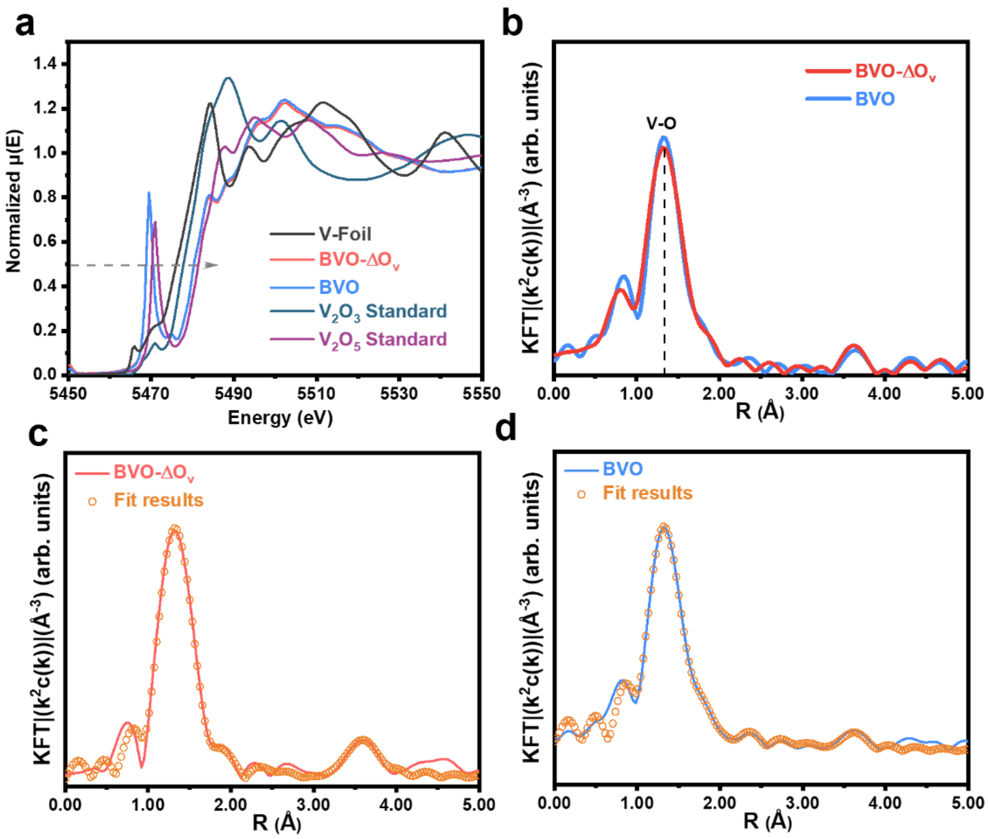

Supplementary Fig. 15. XANES spectra at V *K*-edge of the samples for oxygen vacancy analysis. a Normalized V *K*-edge XANES μ(*E*) spectra, and (**b**) V *K*-edge radial distance χ(*R*) space spectra of BVO-ΔO_v and BVO. Fourier-transformed (FT)-Extended X-ray absorption fine structure (EXAFS) fitting curves at *R* space of V *K*-edge of (**c**) BVO-ΔO_v and (**d**) BVO. Source data are provided as a Source Data file.

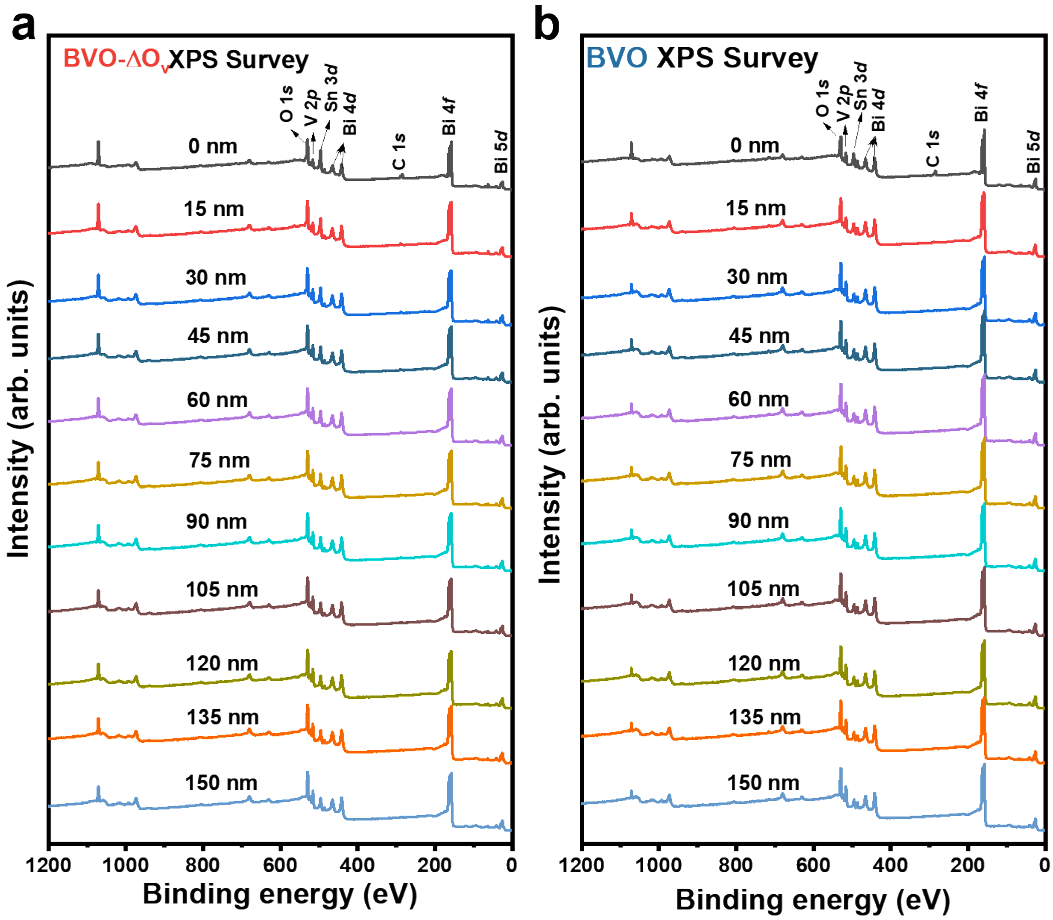

Supplementary Fig. 16. Etching-XPS survey spectra with the etching depth from 0 to 150 nm. a BVO-ΔO_V. **b** BVO. Source data are provided as a Source Data file.

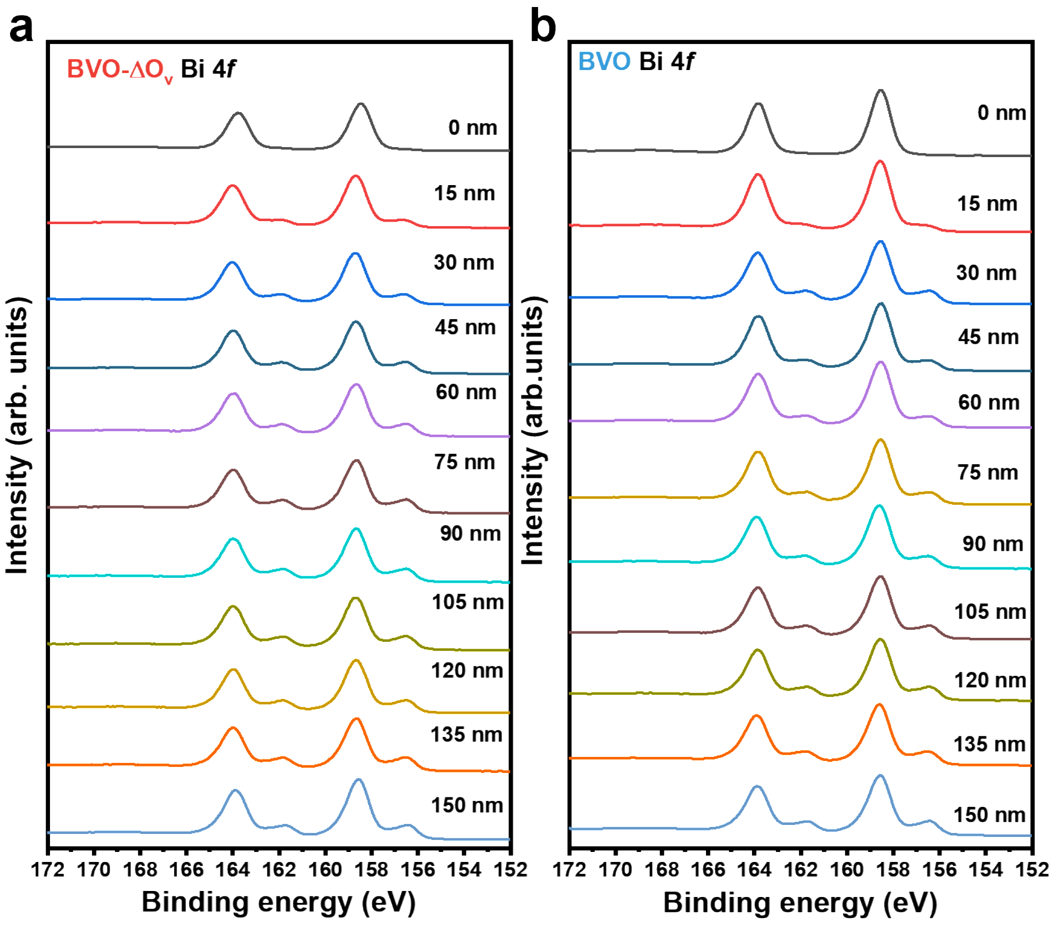

Supplementary Fig. 17. Etching-XPS Bi 4*f* spectra with the etching depth from 0 to 150 nm. a BVO-ΔO_v. **b** BVO. Source data are provided as a Source Data file.

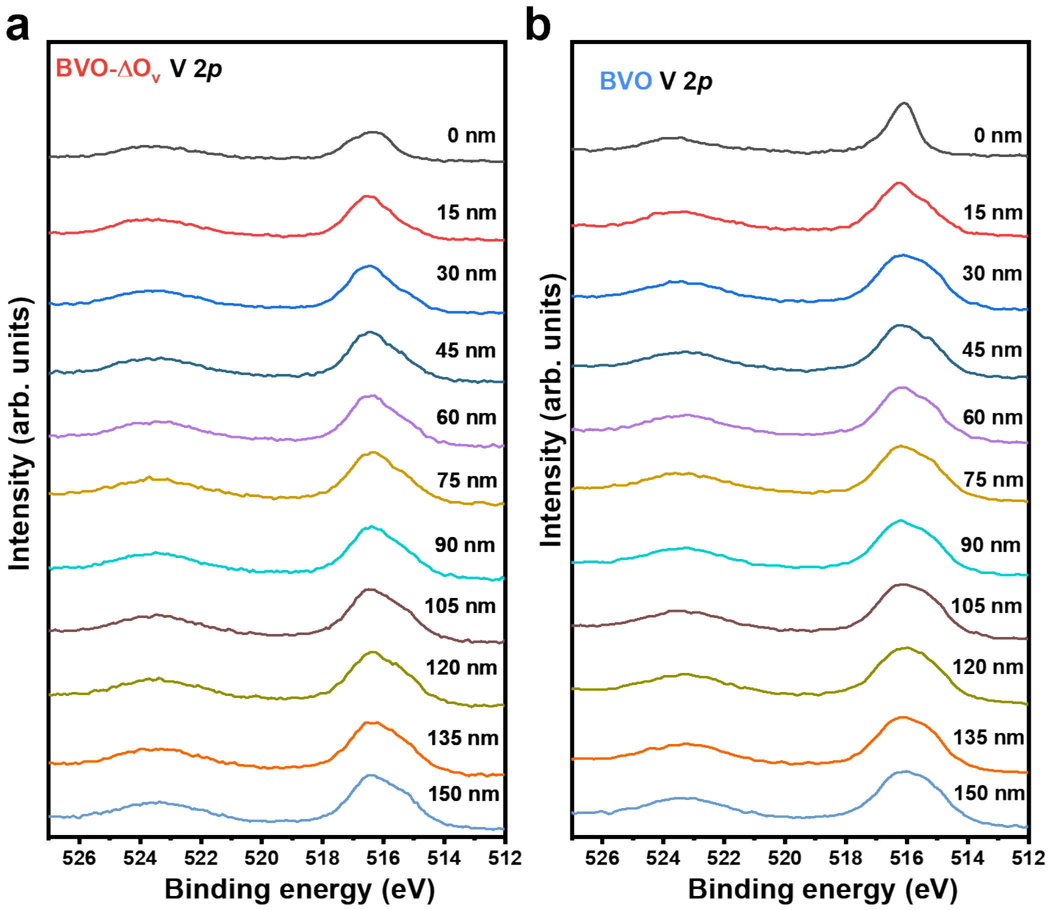

Supplementary Fig. 18. Etching-XPS V 2*p* spectra with the etching depth from 0 to 150 nm. a BVO-ΔOv. **b** BVO. Source data are provided as a Source Data file.

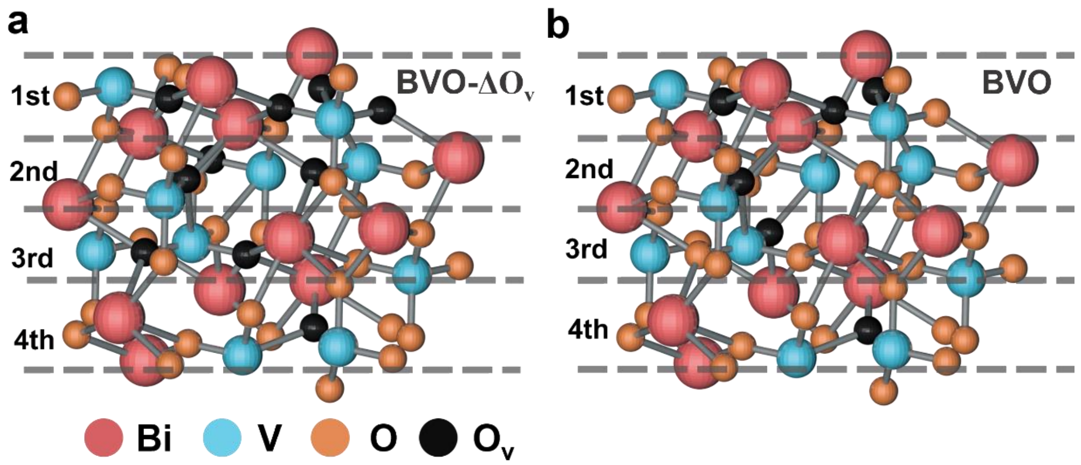

Supplementary Fig. 19. Construction models of the samples. a BVO-ΔOv. **b** BVO.

Supplementary Discussion

To confirm the importance of gradient distributed oxygen vacancies in promoting the charge separation of $BiVO_4$, another BVO-ΔO_v photoanode was annealed in the flow of oxygen at 500 °C for 2 h to remove the oxygen vacancies within the film, and the obtained sample was denoted as BVO-ΔO_v-annealed. Supplementary Fig. 20a shows a much weaker O_v peak in the O 1*s* spectrum of BVO-ΔO_v-annealed compared to that of its BVO-ΔO_v counterpart, suggesting the significant decrease of oxygen vacancies. As expected, the photocurrent density of BVO-ΔO_v-annealed drops significantly for PEC sulfite oxidation (Supplementary Fig. 20b), suggesting the critical role of gradient distributed oxygen vacancies in enhancing charge separation in the bulk of the photoanode.

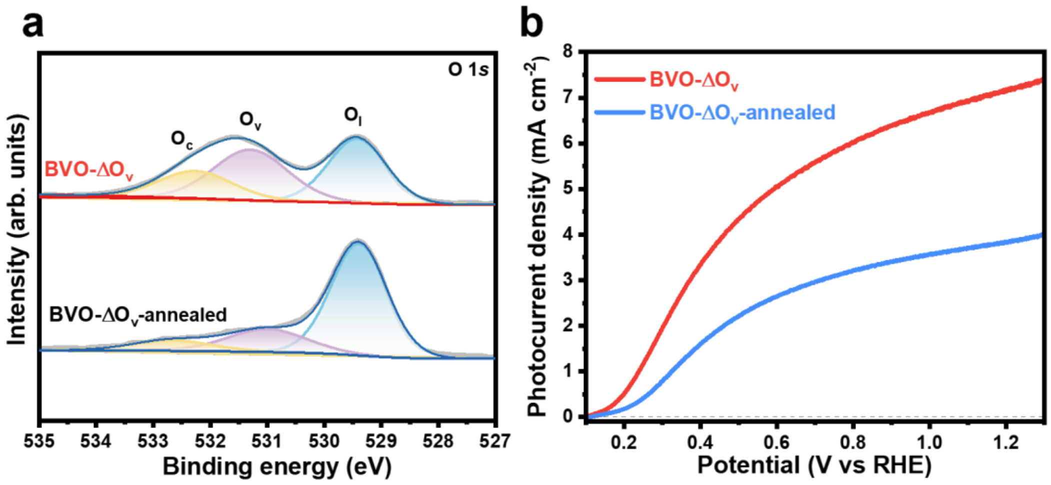

Supplementary Fig. 20. Oxygen vacancies analysis and PEC performance of the samples. a O 1*s* XPS spectra of BVO-ΔO_v and BVO-ΔO_v-annealed. **b** Photocurrent density versus potential curves of BVO-ΔO_v and BVO-ΔO_v-annealed in a 1 M borate buffer electrolyte with 0.1 M Na_2SO_3 (pH 9.5) under AM 1.5G illumination. Source data are provided as a Source Data file.

Supplementary Discussion

The BVO-ΔO_v samples were decorated by FeOOH, NiOOH, NiFeOOH and NiFeCoOOH OECs using a photo-assisted electrodeposition process, and the obtained samples were denoted as BVO-ΔO_v/FeOOH, BVO-ΔO_v/NiOOH, BVO-ΔO_v/NiFeOOH, and BVO-ΔO_v/NiFeCoOOH, respectively. As shown in Supplementary Fig. 21a, the photocurrent densities of the BVO-ΔO_v/FeOOH, BVO-ΔO_v/NiOOH, BVO-ΔO_v/NiFeOOH, and BVO-ΔO_v/NiFeCoOOH samples at 1.23 V vs. RHE are 7.0, 6.6, 6.8, and 7.1 mA cm^{-2}, respectively. Although the BVO-ΔO_v/NiFeCoOOH sample exhibits a slightly higher photocurrent density compared to BVO-ΔO_v/FeOOH, the photocurrent density of BVO-ΔO_v/NiFeCoOOH decreases from 6.6 to 4.6 mA cm^{-2} with a retention rate of 69.6% after 13 h of consecutive AM 1.5G illumination at 1.23 V vs. RHE (Supplementary Fig. 21b). Since the NiFeCoOH OEC contains different metal elements of Ni, Fe and Co, it is challenging to obtain the NiFeCoOH OEC with exactly the same Ni: Fe: Co ratios in different batches. In addition, the photocorrosion of any element in the NiFeCoOH OEC will cause the instability of the surface structure. Therefore, the long-term stability of BVO-ΔO_v/NiFeCoOOH is not good.

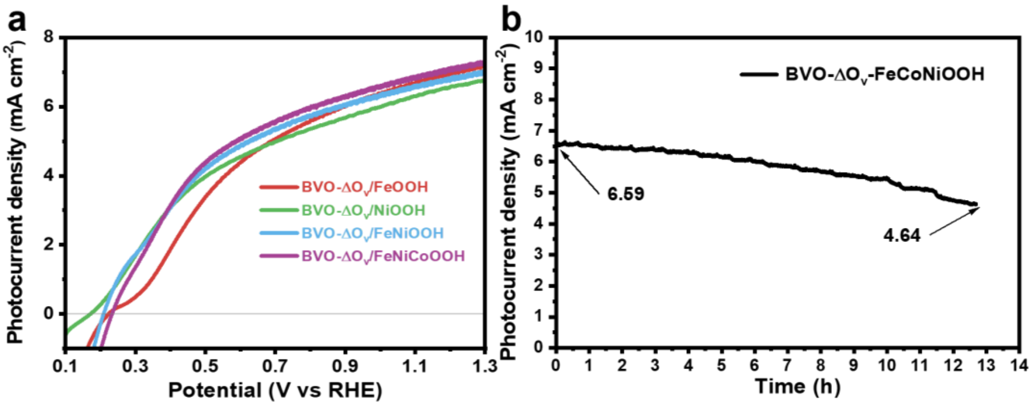

Supplementary Fig. 21. PEC performance of the samples with different OECs. a LSV curves of the BVO-ΔO$_V$/FeOOH, BVO-ΔO$_V$/NiOOH, BVO-ΔO$_V$/NiFeOOH, and BVO-ΔO$_V$/NiFeCoOOH samples. **b** *J*–*t* curve of BVO-ΔO$_V$/NiFeCoOOH at 1.23 V vs. RHE. All measurements are in a 1 M borate buffer electrolyte (pH 9.5) under AM 1.5G illumination. Source data are provided as a Source Data file.

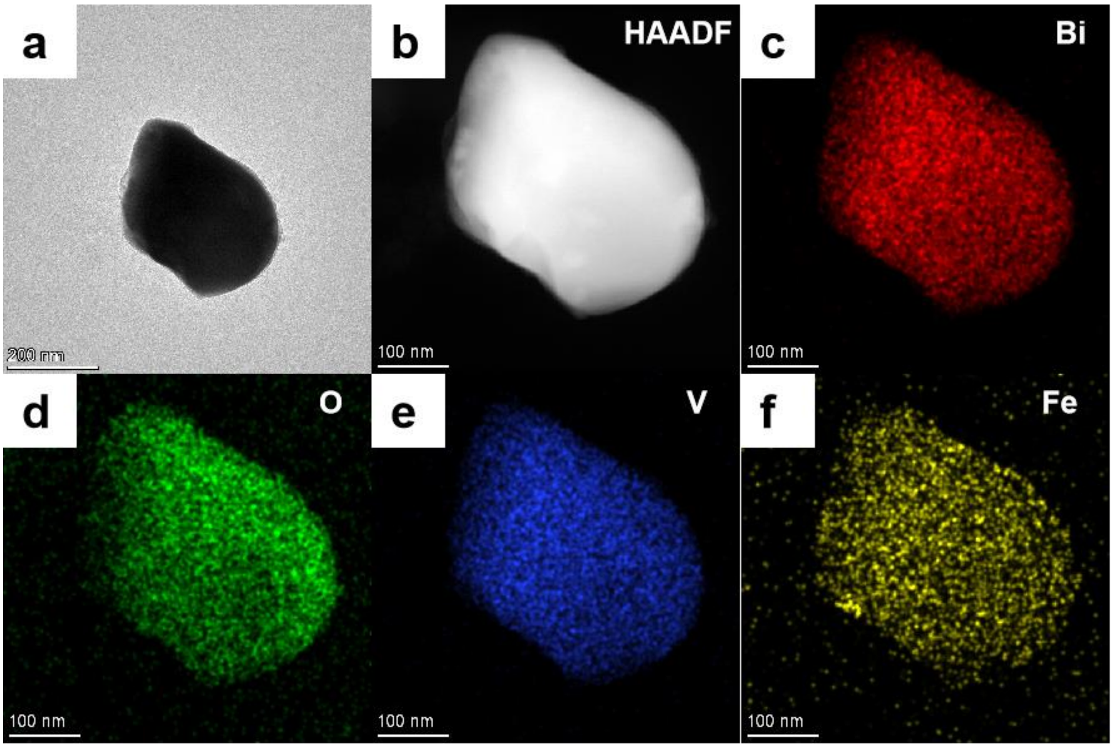

Supplementary Fig. 22. Element distribution analysis of BVO-ΔO_v/FeOOH. a TEM image, (**b**) HAADF image, and EDS mappings of (**c**) Bi, (**d**) O, (**e**) V, and (**f**) Fe of BVO-ΔO_v/FeOOH.

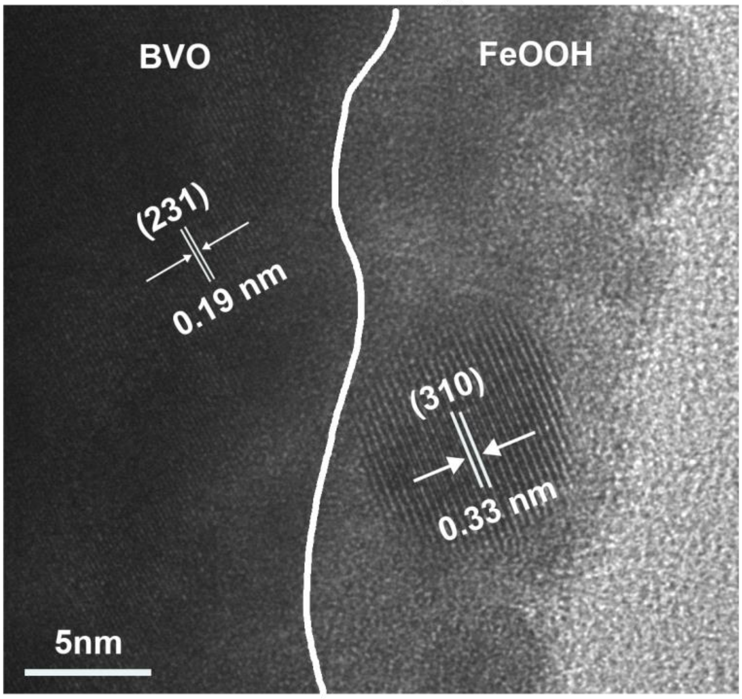

Supplementary Fig. 23. Composite analysis. HRTEM image of BVO-ΔO_v/FeOOH.

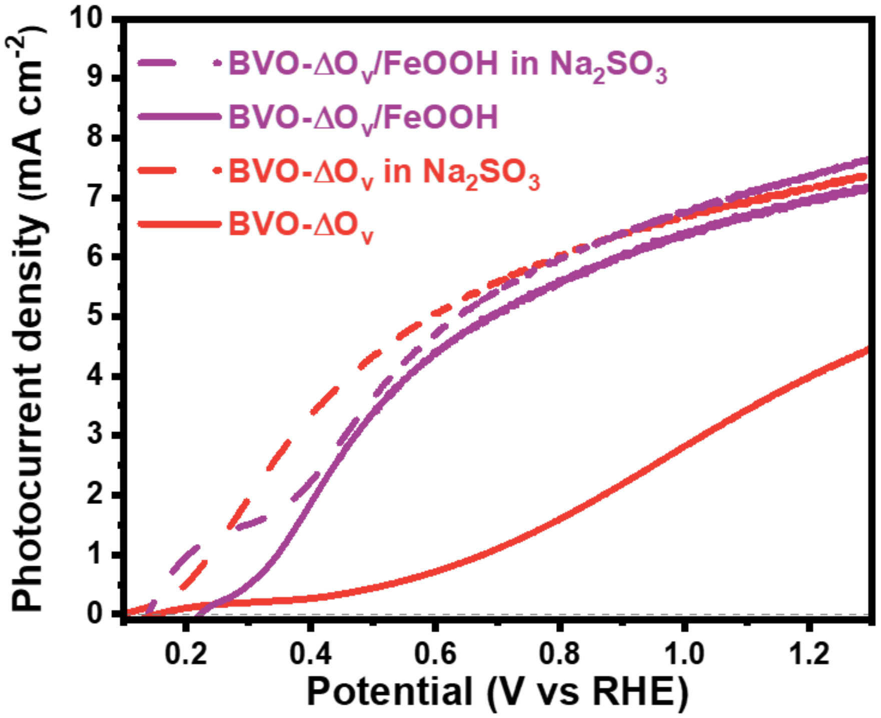

Supplementary Fig. 24. PEC performance of the samples. Photocurrent density versus potential curves of BVO-ΔOv and BVO-ΔOv/FeOOH in a 1 M borate buffer electrolyte with and without 0.1 M Na_2SO_3 (pH 9.5) under AM 1.5G illumination. Source data are provided as a Source Data file.

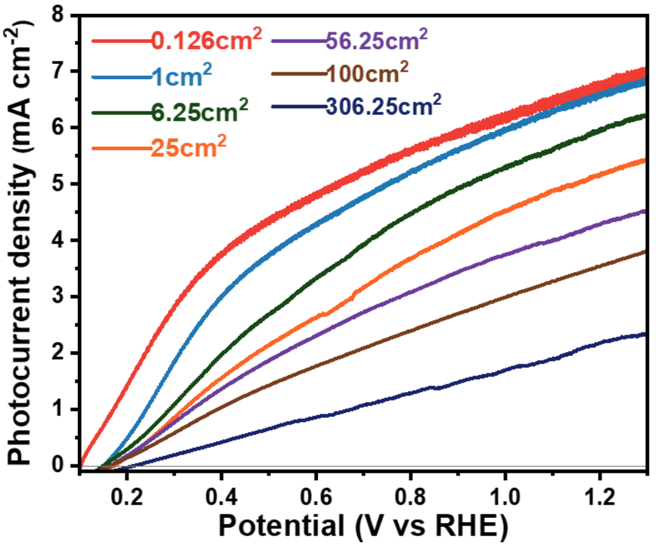

Supplementary Fig. 25. Analysis of the geometrical area effect on the photocurrent density. Photocurrent density versus potential curves of BVO-ΔO_v/FeOOH photoanodes with different exposed areas in a 1 M borate buffer electrolyte (pH 9.5) under AM 1.5G illumination. Source data are provided as a Source Data file.

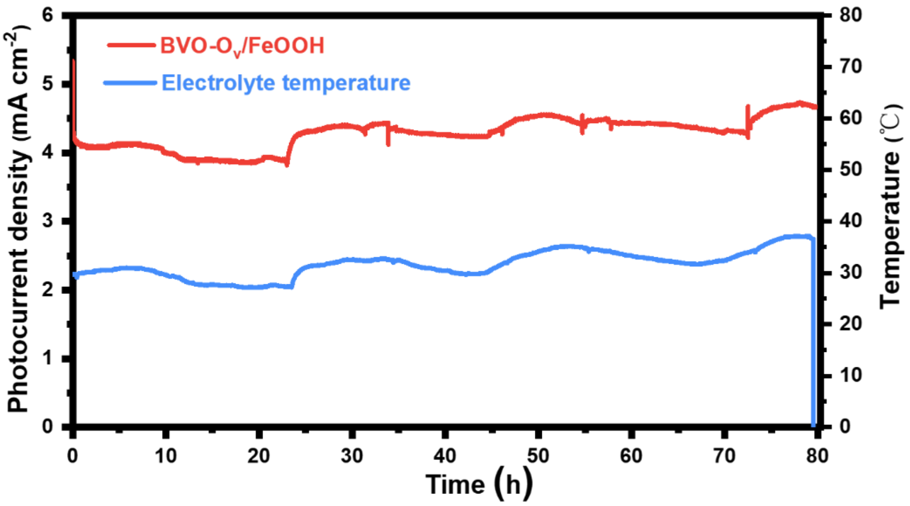

Supplementary Fig. 26. Effect of the temperature on the photocurrent density. J-t curve of a BVO-ΔO_v/FeOOH photoanode in a 1 M borate buffer electrolyte (pH 9.5) under AM 1.5G illumination and the change of temperature in the electrolyte. Source data are provided as a Source Data file.

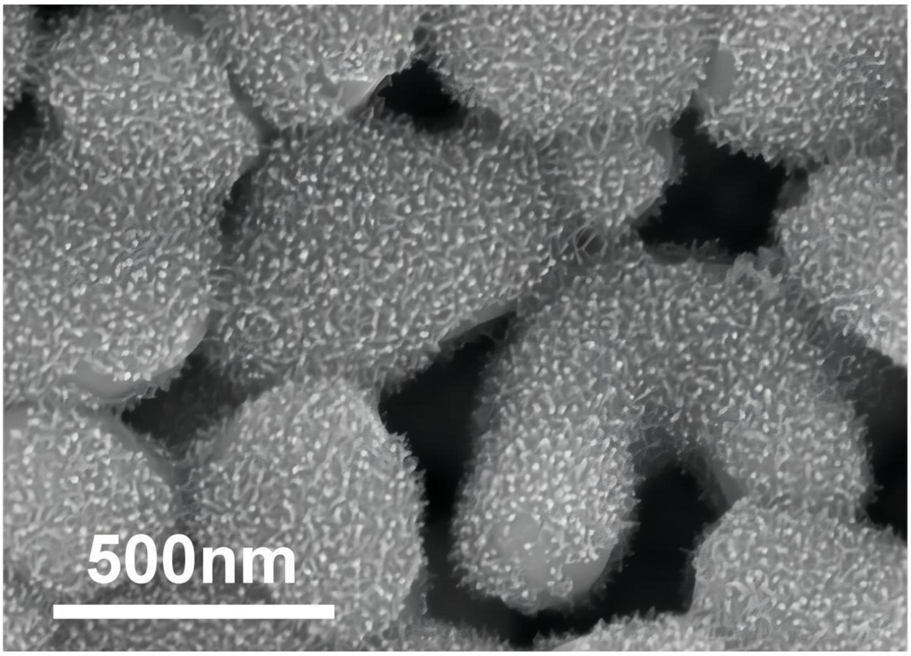

Supplementary Fig. 27. Morphology characterization after stability test. SEM image of BVO-ΔO_V/FeOOH after 500 h of stability test at 0.6 V vs. RHE.

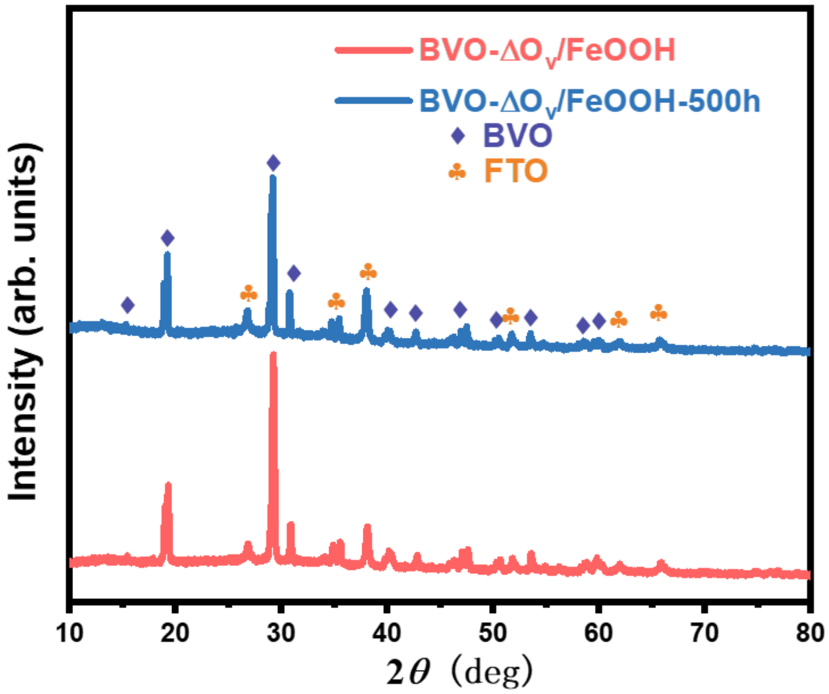

Supplementary Fig. 28. Crystal structure characterization after stability test. XRD patterns of BVO-ΔO_V/FeOOH before and after 500 h of stability test at 0.6 V vs. RHE. Source data are provided as a Source Data file.

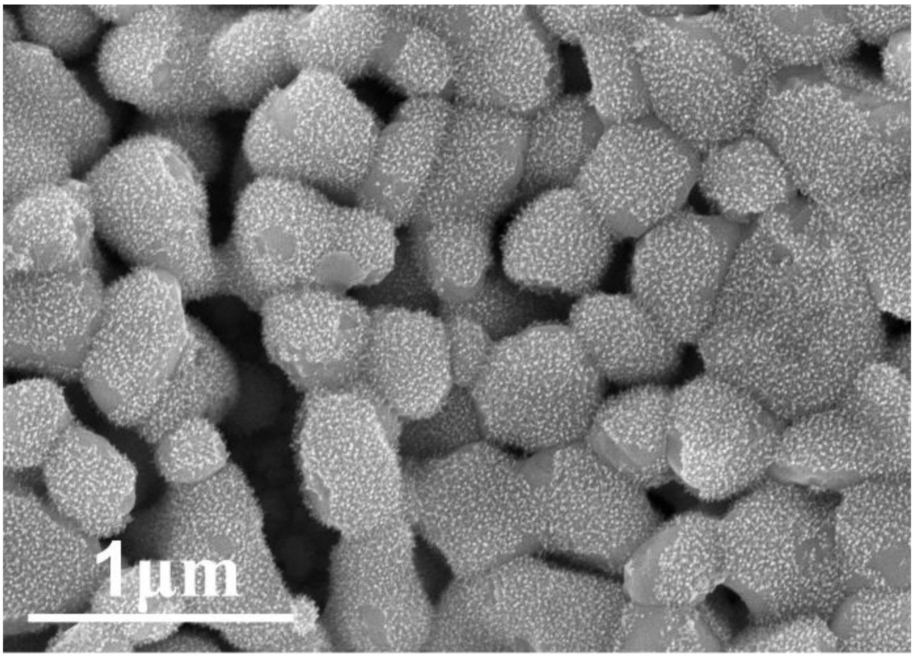

Supplementary Fig. 29. Morphology characterization after stability test. SEM image of BVO-ΔO_v/FeOOH after 540 h of stability test at 0.6 V vs. RHE.

Supplementary Discussion

The long-term stability performance of a BVO-ΔO_v/FeOOH film for PEC water splitting was also measured at 1.23 V vs. RHE under consecutive AM 1.5G illumination. As shown in Fig. 4g, more fluctuations of the photocurrent densities can be observed compared to the measurement at 0.6 V vs. RHE, which is attributed to the evolution of much more oxygen bubbles from the photoanode. A stable photocurrent density of 6.8 mA cm^{-2} is observed until around 470 h. Gradual decay of the photocurrent density can be observed in the range of 470-520 h, decreasing to 4.9 mA cm^{-2} at 520 h.

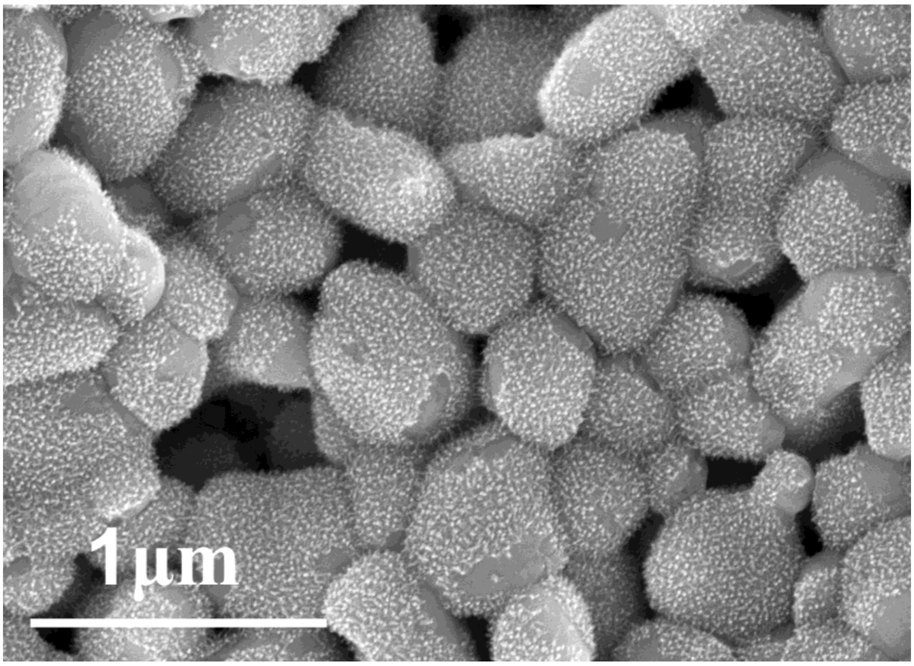

Supplementary Fig. 30. Morphology characterization after stability test. SEM image of BVO-ΔO_v/FeOOH after 520 h of stability test at 1.23 V vs. RHE.

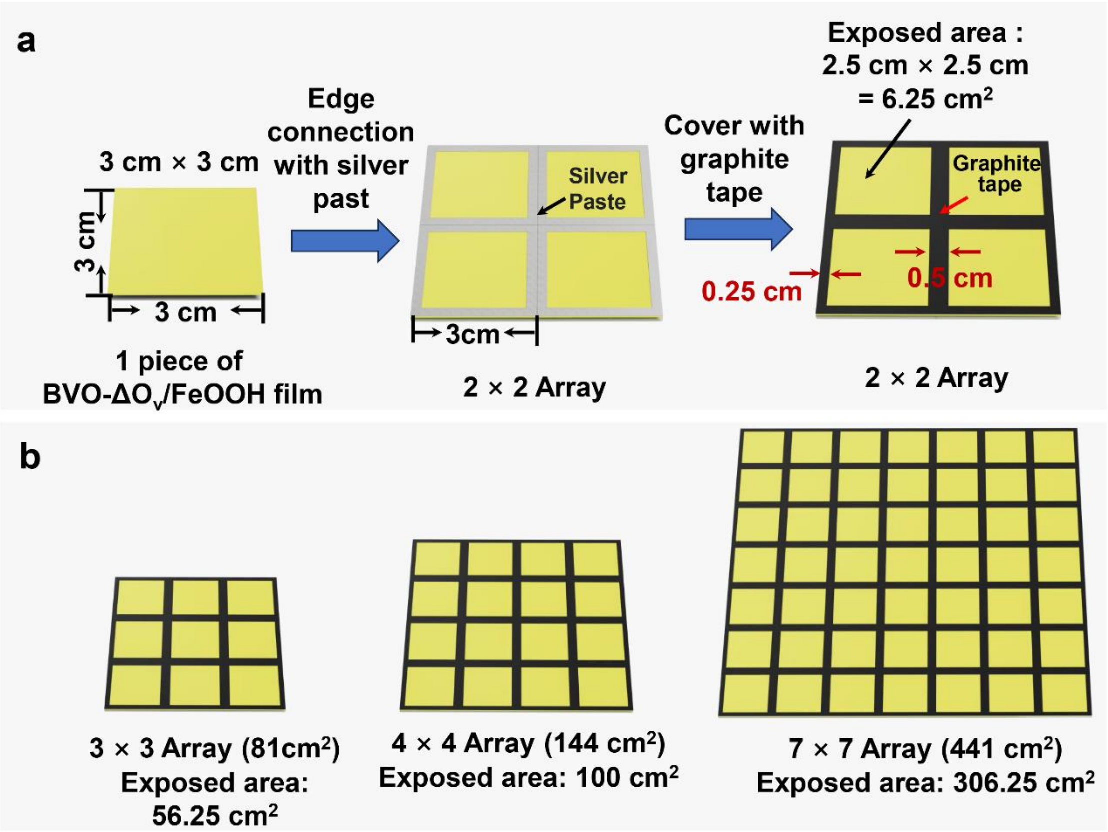

Supplementary Fig. 31. Scheme of the assembly of large-area photoanodes. a An example of assembling a 2 × 2 array BVO-ΔO_V/FeOOH photoanode (6 cm × 6 cm). **b** schemes of 3 × 3 (9 cm × 9 cm), 4 × 4 (12 cm × 12 cm), and 7 × 7 (21 cm × 21 cm) array BVO-ΔO_V/FeOOH photoanodes.

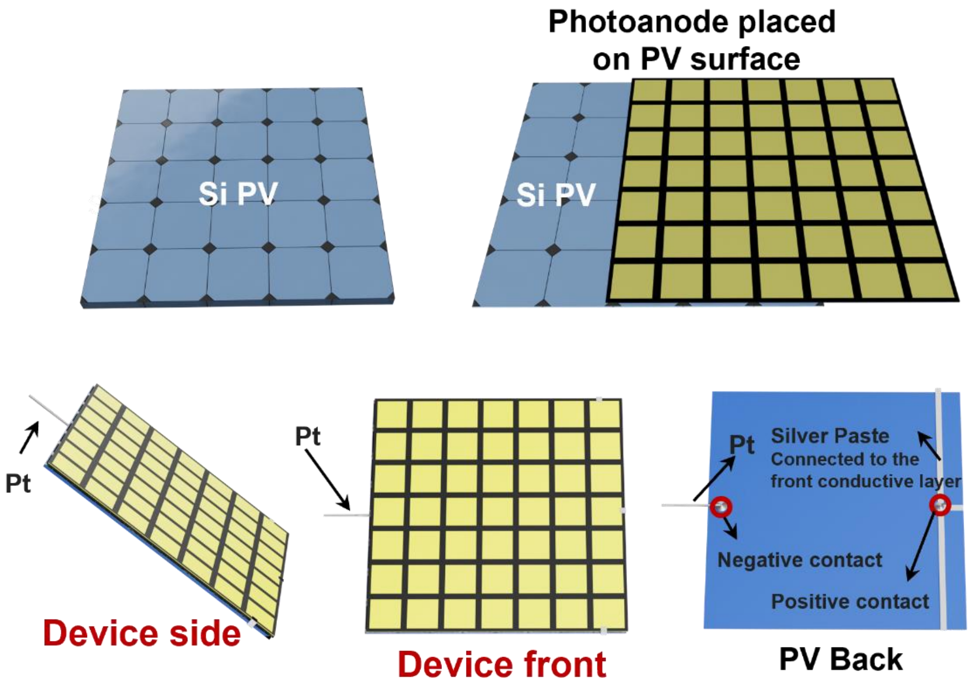

Supplementary Fig. 32. Scheme of the assembly of a 7 × 7 array artificial leaf. Illustration of a 21 cm × 21 cm Si PV panel, a BVO-ΔO_V/FeOOH photoanode (21 cm × 21 cm), and different views of the artificial leaf.

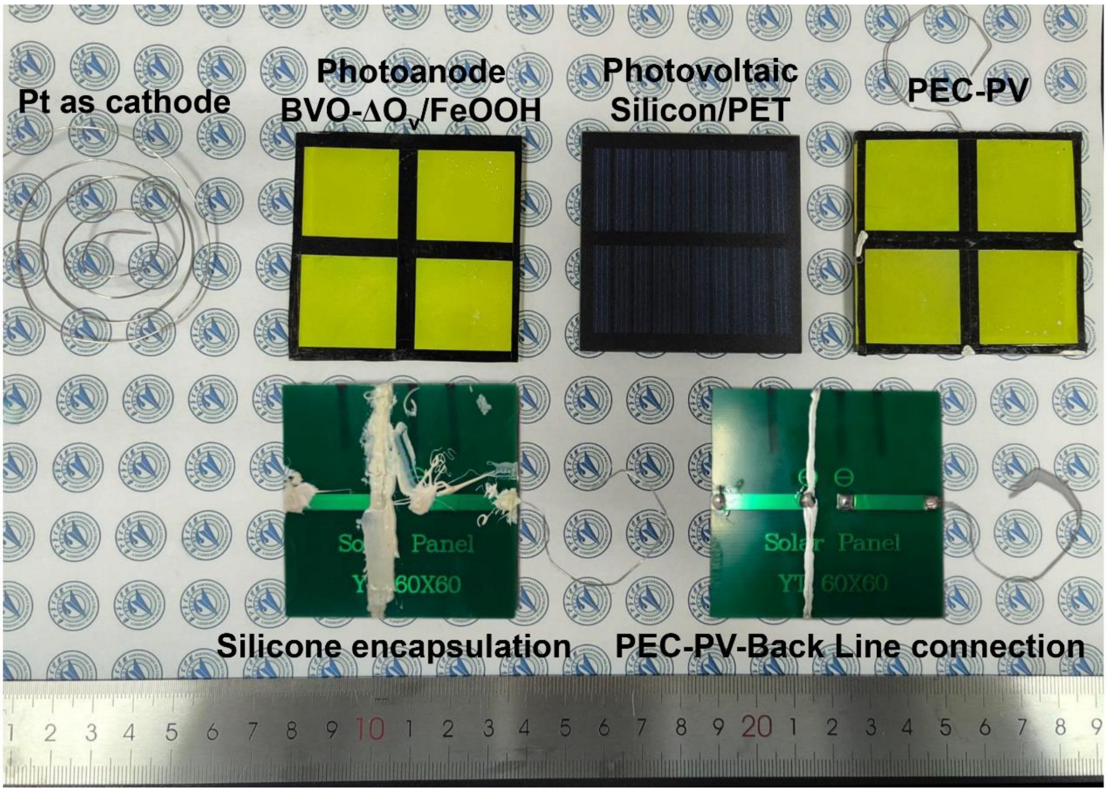

Supplementary Fig. 33. The structure of a 2 × 2 array artificial leaf. Digital images of a 2 × 2 (6 cm × 6 cm) array artificial leaf with different components.

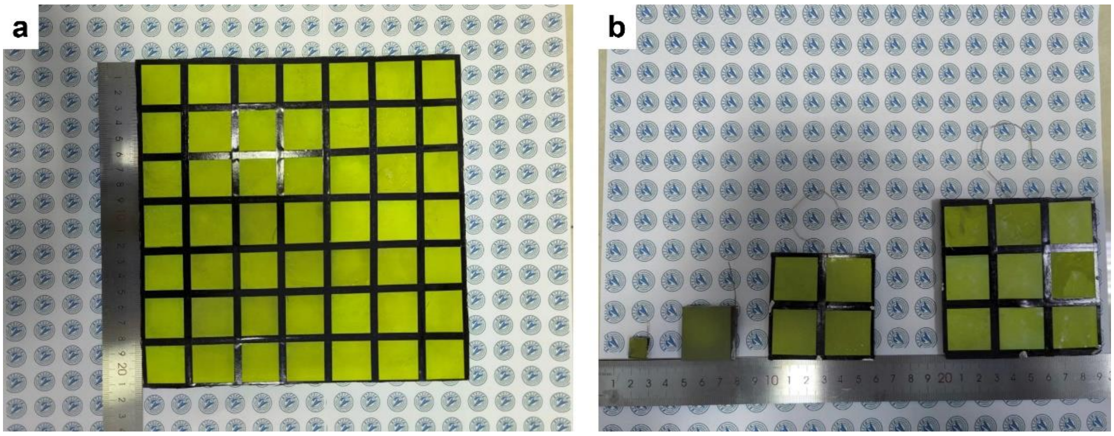

Supplementary Fig. 34. Artificial leaves with different sizes. Digital images of (**a**) a 21 cm × 21 cm artificial leaf, and (**b**) artificial leaves with different dimensions of 1 cm × 1 cm, 3 cm × 3 cm, 6 cm × 6 cm, and 9 cm × 9 cm.

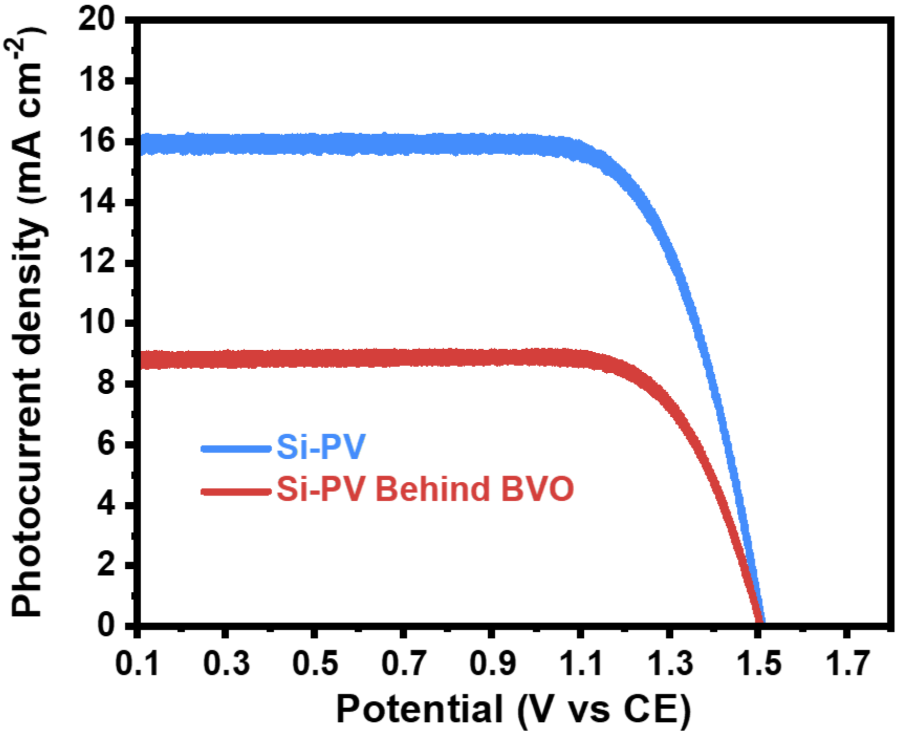

Supplementary Fig. 35. Performance of Si PVs. J-V curves of Si PVs with and without the surface covered by a BVO-ΔO_v/FeOOH photoanode. Source data are provided as a Source Data file.

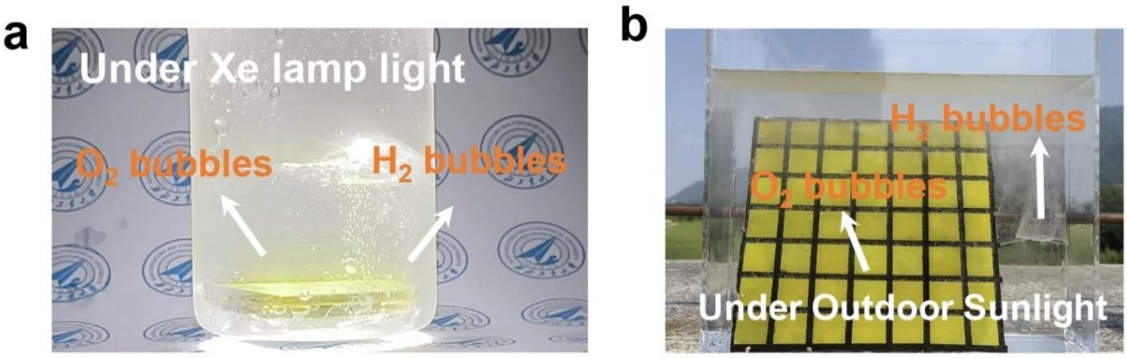

Supplementary Fig. 36. Digital images of water splitting from wireless artificial leaves with different sizes. a A wireless BVO-ΔO_v/FeOOH-PV artificial leaf with dimensions of 3 cm × 3 cm for water splitting under Xe lamp light (100 mW cm^{-2}). **b** A wireless $BiVO_4$-PV artificial leaf with dimensions of 21 cm × 21 cm (exposed area: 306.25 cm^2) for water splitting under natural sunlight.

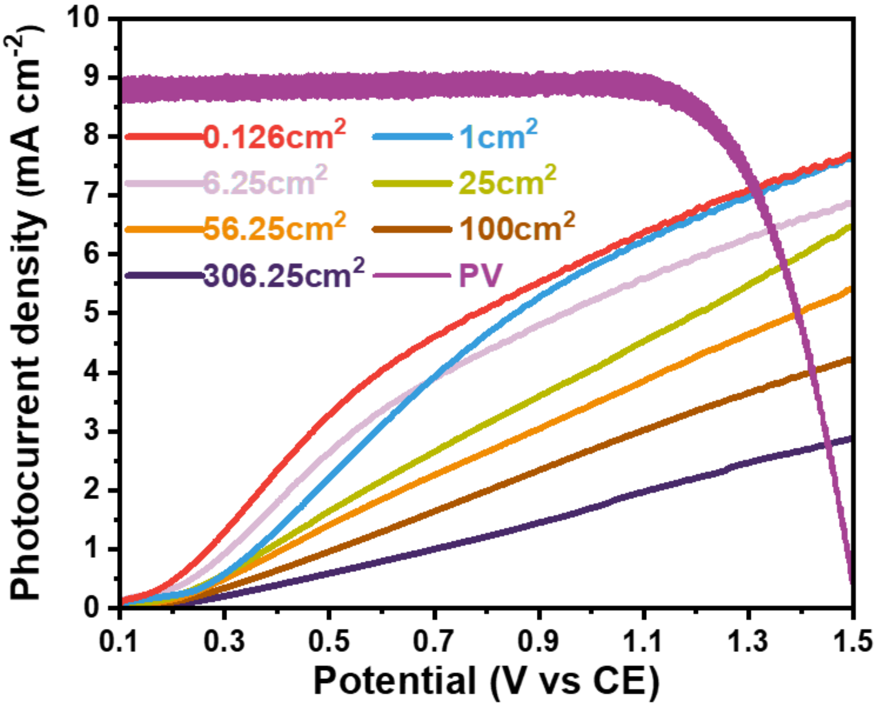

Supplementary Fig. 37. STH efficiencies of BVO-ΔO_v/FeOOH-PV artificial leaves with different sizes. J–V curves of a BVO-ΔO_v/FeOOH photoanode and PV behind the BVO-ΔO_v/FeOOH photoanode in tandem with different exposed areas of 0.126, 1, 6.25, 25, 56.25, 100, and 306.25 cm^2 under AM 1.5 G irradiation. Source data are provided as a Source Data file.

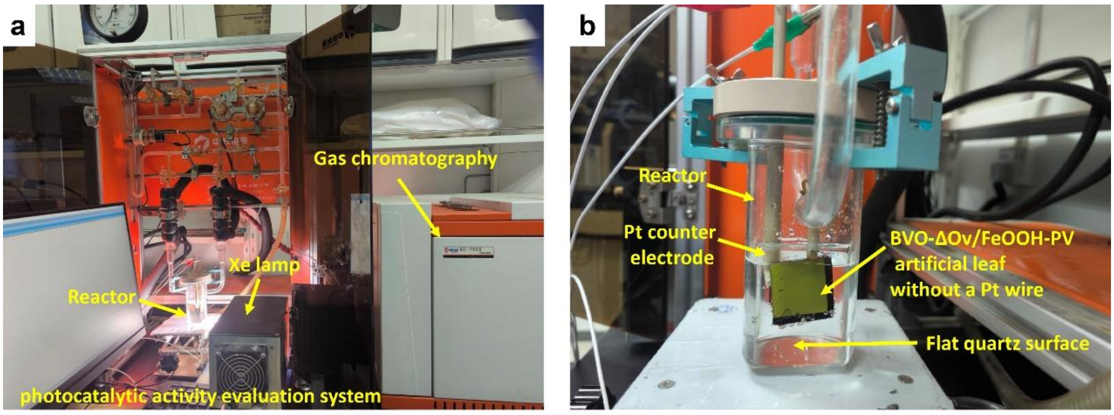

Supplementary Fig. 38. Gas evolution measurement for a wired artificial leaf. a Digital image of gas evolution measurement for a wired artificial leaf connected with a gas chromatography. **b** Digital image of a reactor for measuring the gas evolution and photocurrent densities of a wired artificial leaf. The reactor can measure gas evolution of a wired artificial leaf with dimensions up to 3 cm × 3 cm.

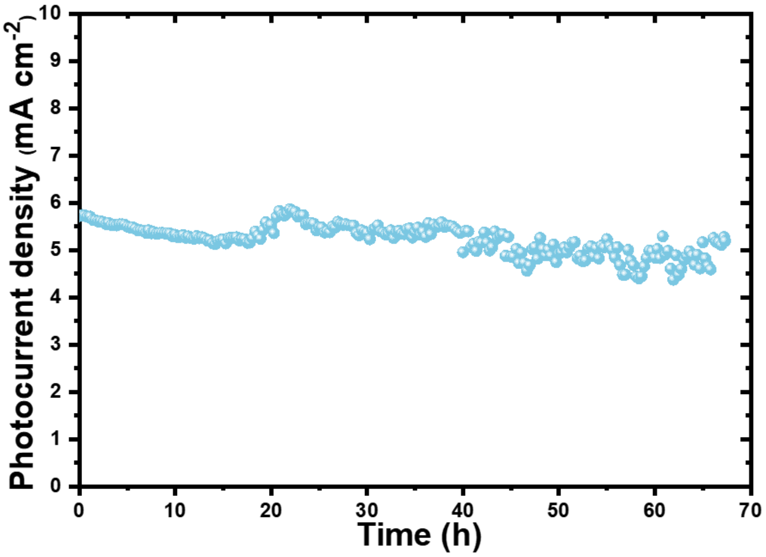

Supplementary Fig. 39. Stability measurement of a wired artificial leaf with imperfect encapsulation of the connections. J-V curve of a wired BVO-ΔO_v/FeOOH-PV wired artificial leaf with dimensions of 3 cm × 3 cm. Source data are provided as a Source Data file.

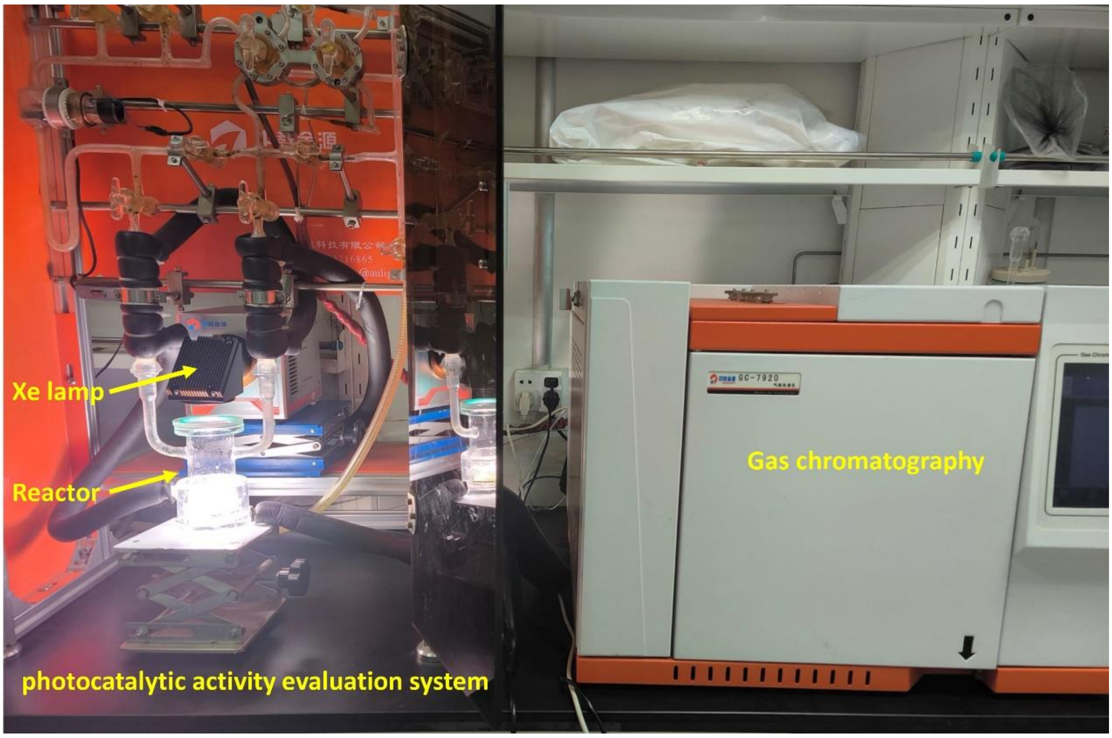

Supplementary Fig. 40. Gas evolution measurement for a wireless artificial leaf. Digital image of gas evolution measurement for a wireless artificial leaf in a photocatalytic activity evaluation system connected with a gas chromatography. The reactor can measure gas evolution of a wireless artificial leaf with dimensions up to 3 cm × 3 cm.

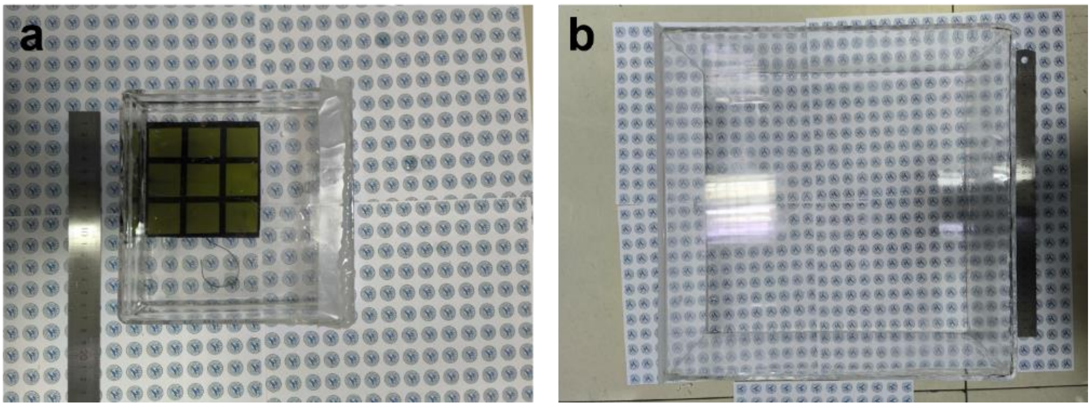

Supplementary Fig. 41. Gas evolution measurement for a wireless artificial leaf. Digital images of (**a**) a sealed quartz reactor with dimensions of 15 cm × 15 cm × 10 cm (inside is a wireless artificial leaf with dimensions of 9 cm × 9 cm as reference), and (**b**) a sealed quartz reactor with dimensions of 30 cm × 30 cm × 10 cm.

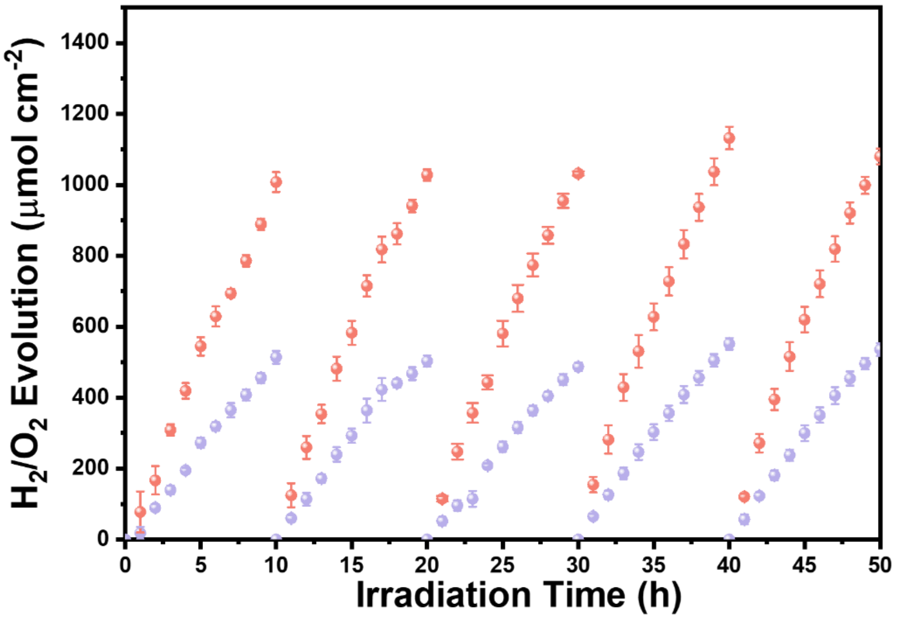

Supplementary Fig. 42. Gas evolution performance for a wireless artificial leaf. Average H_2 and O_2 evolution performances of 3 artificial leaves with dimensions 3 cm × 3 cm (exposed area 6.25 cm^2). Error bars represent standard deviation. Source data are provided as a Source Data file.

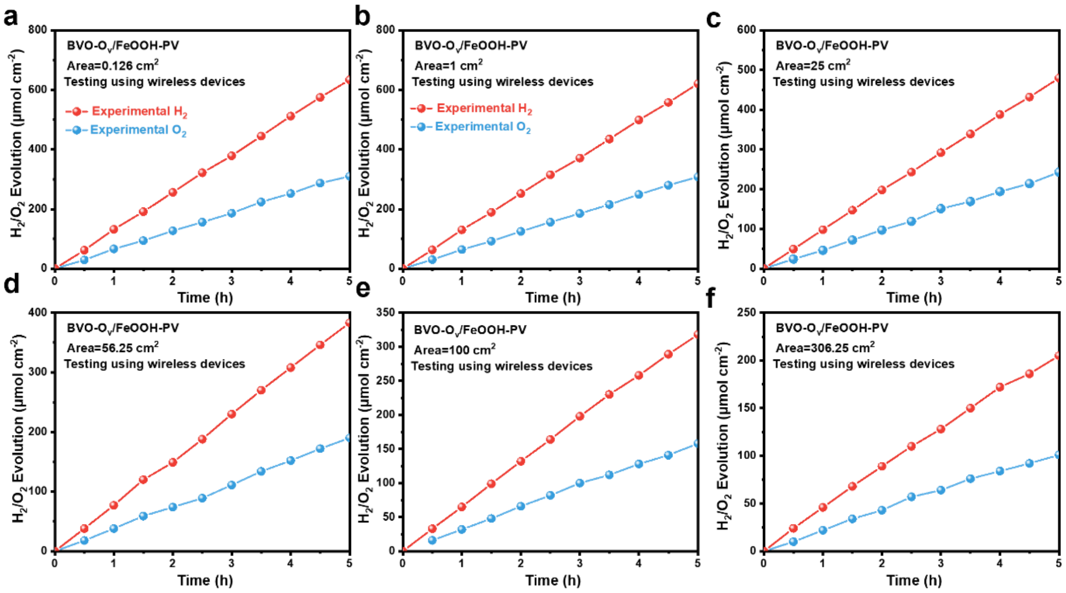

Supplementary Fig. 43. Gas evolution performance for wireless artificial leaves with different sizes. a 1 cm × 1 cm (control exposed area 0.126 cm^2), (**b**) 1 cm × 1 cm (exposed area: 1 cm^2), (**c**) 6 cm × 6 cm (exposed area: 25 cm^2), (**d**) 9 cm × 9 cm (exposed area: 56.25 cm^2), (**e**) 12 cm × 12 cm (exposed area: 100 cm^2) and (**f**) 21 cm × 21 cm (exposed area: 306.25 cm^2). Source data are provided as a Source Data file.

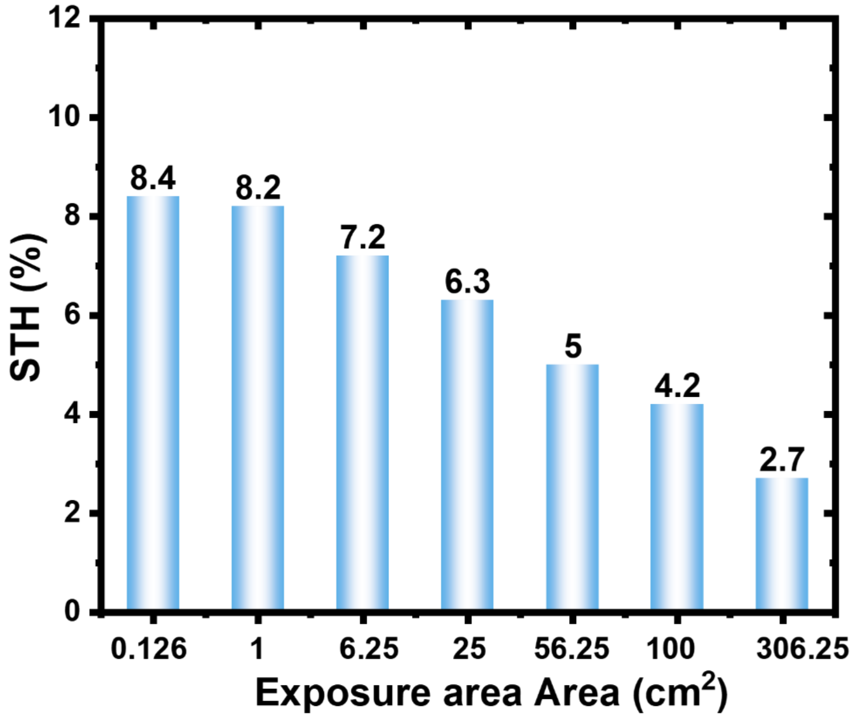

Supplementary Fig. 44. STH efficiencies for wireless artificial leaves with different sizes. STH efficiencies of wireless artificial leaves with different sizes calculated based on their hydrogen evolution performances: 1 cm × 1 cm (control exposed area 0.126 cm^2), 1 cm × 1 cm (exposed area: 1 cm^2), 3 cm × 3 cm (exposed area 6.25 cm^2), 6 cm × 6 cm (exposed area: 25 cm^2), 9 cm × 9 cm (exposed area: 56.25 cm^2), 12 cm × 12 cm (exposed area: 100 cm^2) and 21 cm × 21 cm (exposed area: 306.25 cm^2). Source data are provided as a Source Data file.

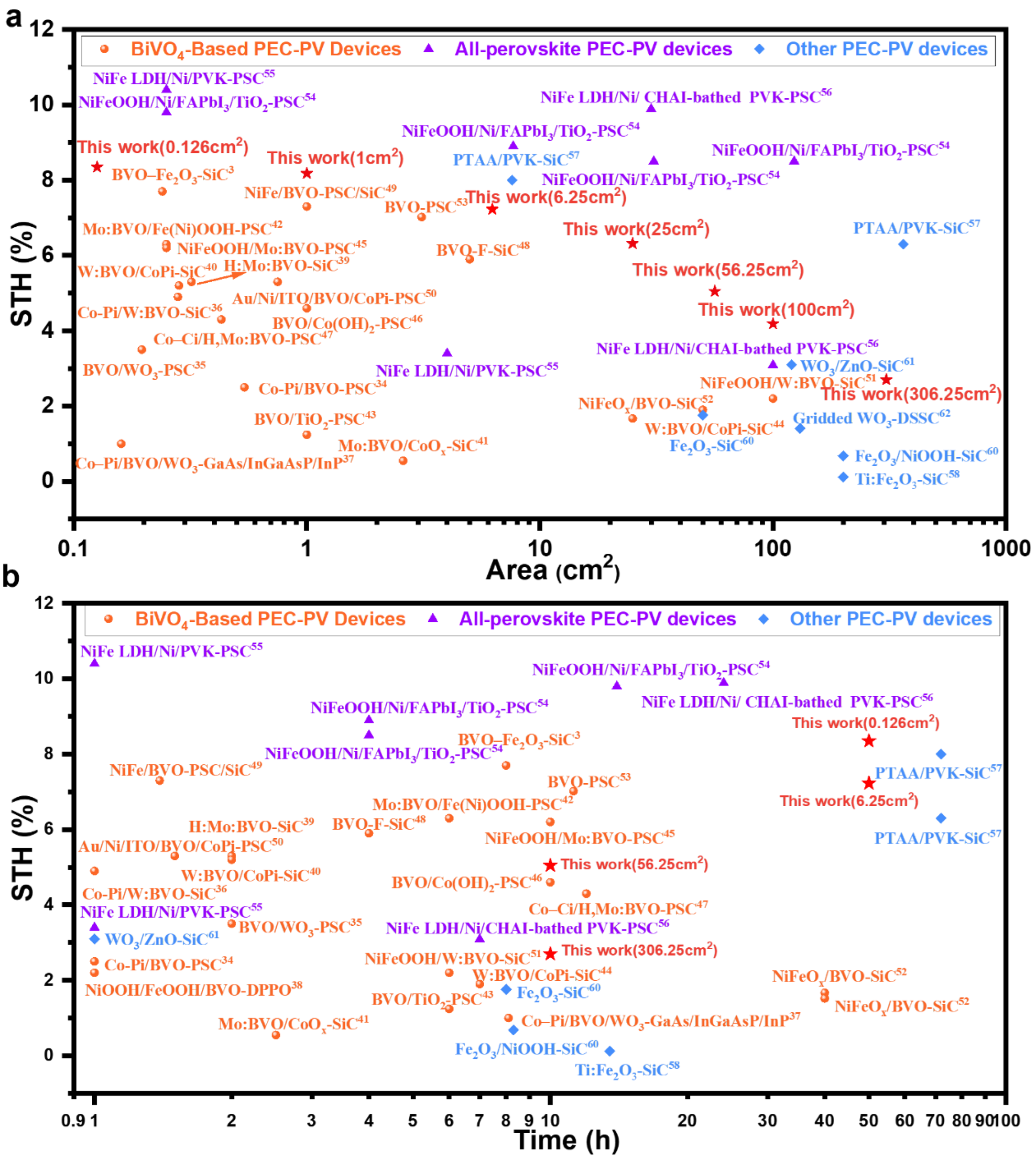

Supplementary Fig. 45. Comparison of STH efficiencies with other devices. a STH efficiency vs. area and (**b**) STH efficiency vs. operation time for $BiVO_4$-based PEC-PV devices, all perovskite PEC-PV devices, and other PEC-PV devices. (Detailed information is listed in Supplementary Table 9).

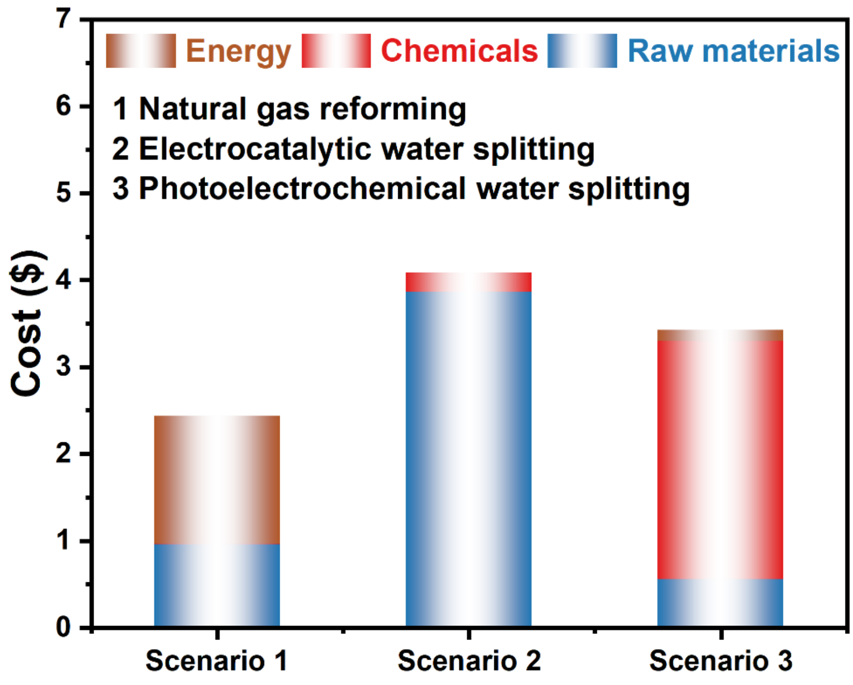

Supplementary Fig. 46. Techno-economic comparison of different technologies for hydrogen production. Comparison for the cost of producing 1 kg of hydrogen from three different technologies: natural gas reforming (Scenario 1), electrocatalytic water splitting (Scenario 2), and photoelectrochemical water splitting (Scenario 3). Source data are provided as a Source Data file.

Supplementary Table 1. Comparison of our photoanode to other $BiVO_4$/OEC photoanodes.

Photoanodes	Photocurrent density (mA cm^{-2} at 1.23V vs. RHE)	ABPE(%)	Stability/h	Ref.
BVO@ZCF(P)-O	5.7	2.20	40	17
MOD-BVO/$NiFeO_x$	4.2	1.53	27	18
P-$BiVO_4$/$NiFeO_x$	4.08 (0.6V)	2.21	10	19
$BiVO_4$/$FeNiPO_x$	6.73	-	84	20
NiFeOOH/MoO_x/MQD/BVO	5.85	2.43	100	21
FeNiOOH–LGCDs-PHGs–MBVO	6.08	-	120	22
BVO/N:$NiFeO_x$	6.4	1.9	5	23
BVO/Ni-N_4-O/FeOOH	6.0	2.66	20	7
NiOOH/FeOOH/Co_3O_4/BVO	6.34	2.72	4	24
O_V-$BiVO_4$@NiFe-MOFs	5.3	1.62	10	25
O_V-$BiVO_4$/$NiFeO_x$	5.54	1.85	100	26
$BiVO_4$/FeOOH/NiOOH	4.8	1.75	50	13
E-$BiVO_4$/BPQDs/OL-OEC	6.2	-	50	27
NiFe-OEC/Mo:$BiVO_4$/Ni/Sn	3.7	1.6	1100	28
$BiVO_4$/FeOOH/NiOOH in KB+Vsolution	4.7	-	500	29
Co‐Pi/BVO‐VO	5.0	1.55	-	30
N_2-treated $BiVO_4$/FeOOH/NiOOH	5.0	2.2	50	31
NiOOH/BP/$BiVO_4$	4.48	-	60	32
$NiFeO_x$/B-C_3N_4/Mo-$BiVO_4$	5.93(±0.3)	2.67	10	33
**BVO-ΔO_v/FeOOH**	**7.0**	**2.78**	**520**	**This work**

Supplementary Table 2. Fitted results of the EIS curves of BVO-ΔO_v and BVO in Fig. 2f.

Samples	R_{ct}/ Ω
BVO-ΔO_v	3121
BVO	4163

Supplementary Table 3. Carrier densities (N_d) of BVO-ΔO_v and BVO.

Samples	N_d/10^{17} cm^{-3}
BVO-ΔO_v	11.0
BVO	6.55

Supplementary Table 4. Average surface potential of BVO-ΔO_v and BVO under light and dark.

Samples	P_{avg}-light (mV)	P_{avg}-dark (mV)	ΔP_{avg} (mV)
BVO-ΔO_v	206.8	105.5	101.3
BVO	179.3	164.5	14.80

Supplementary Table 5. Fitting parameters for the TRPL curves of the BVO-ΔO_v and BVO films.

Samples	τ_{avg} (ns)	τ_1 (ns)	τ_2 (ns)	A_1	A_2	χ^2
BVO-ΔO_v	42.40	2.649	50.34	207.0	54.56	0.846
BVO	22.83	3.206	35.05	212.7	31.22	1.018

Supplementary Table 6. Fitting parameters of Bi *L*3-edge EXAFS curve for BVO-ΔO_v and BVO.

Samples	Path[a]	R(Å)[b]	N[c]	σ^2(Å^{-2})[f]	ΔE_0 (eV)[g]	Rf, %
BVO-ΔO_v	Bi-O1	2.14 (2.18)	2[e]	0.0002	-18.29	0.77
	Bi-O2	2.29 (2.33)	2[e]	0.008		
	Bi-O3	3.51 (3.51)	2[e]	0.009		
	Bi-V1	3.32 (3.57)	2[e]	0.007		
	B-V2	3.68 (3.68)	3[e]	0.010		
	Bi-Bi	4.36 (4.79)	4[e]	0.000		
BVO	Bi-O1	2.23	2[e]	0.004	-3.14	1.05
	Bi-O2	2.38	2[e]	0.007		
	Bi-O3	3.64	2[e]	0.0008		
	Bi-V1	3.74	2[e]	0.005		
	Bi-V2	3.79	3[e]	0.010		
	Bi-Bi	4.48	4[e]	0.000		

[a] The distances for the path are from the crystal structure of $BiVO_4$ (1101208).

[b] R: average distance between absorber and backscattered atoms.

[c] N: coordination number.

[d e] The coordination number is fixed according to the crystal structure model.

[f] σ^2: Debye-Waller factor.

[g] ΔE_0: the inner potential correction.

The data range used for fitting in R space (ΔR) is 1.0−6.0 Å.

Supplementary Table 7. Fitting parameters of V *K*-edge EXAFS curve for BVO-ΔO_v and BVO.

Samples	Path[a]	R(Å)[b]	N[c]	σ^2(Å^{-2})[f]	ΔE_0 (eV)[g]	Rf, %
BVO	V-O1	1.68 (1.72)	2[e]	0.0006	12.36	0.09
	V-O2	2.29 (1.78)	2[e]	0.0007		
	V-Bi	3.58 (3.58)	2[e]	0.011		
BVO-ΔO_v	V-O1	1.68	2[e]	0.0005	10.95	0.12
	V-O2	1.83	2[e]	0.0005		
	V-Bi	3.57	2[e]	0.012		

[a] The distances for the path are from the crystal structure of $BiVO_4$ (1101208).

[b] R: average distance between absorber and backscattered atoms.

[c] N: coordination number.

[d e] The coordination number is fixed according to the crystal structure model.

[f] σ^2: Debye-Waller factor.

[g] ΔE_0: the inner potential correction.

The data range used for fitting in R space (ΔR) is 1.0−5.0 Å.

Supplementary Table 8. *J*−*V* Parameters of the Si PV panels.

Parameter	Value
J_{sc}	16 mA cm^{-2}
V_{oc}	1.5 V
FF	0.75
PCE	18%

Supplementary Table 9. Comparison of photoanodes combined with photovoltaics for unassisted PEC-PV water splitting systems.

PEC	PV	STH (%)	Stability (h)	Area (cm^2)	Configuration	Year	Ref.
**BVO-ΔO_v/FeOOH**	**c-Si**	**8.4**	**50**	**0.126**	**Tandem**	**This work**	
**BVO-ΔO_v/FeOOH**	**c-Si**	**8.2**	**-**	**1**	**Tandem**	**This work**	
**BVO-ΔO_v/FeOOH**	**c-Si**	**7.2**	**50**	**6.25**	**Tandem**	**This work**	
**BVO-ΔO_v/FeOOH**	**c-Si**	**6.3**	**-**	**25**	**Tandem**	**This work**	
**BVO-ΔO_v/FeOOH**	**c-Si**	**5.0**	**-**	**56.25**	**Tandem**	**This work**	
**BVO-ΔO_v/FeOOH**	**c-Si**	**4.2**	**10**	**100**	**Tandem**	**This work**	
**BVO-ΔO_v/FeOOH**	**c-Si**	**2.7**	**10**	**306.25**	**Tandem**	**This work**	
Co–Pi/BVO	$MAPbI_3$	2.5	1	0.54	Tandem	2015	34
BVO/WO_3/SnO_2	$MAPbI_3$	3.5	2	0.196	1 PV cell	2017	35
Co-Pi/W:BVO	2-jna-Si	4.9	1	0.28	Parallel	2013	36
Co–Pi/BVO/WO-GaAs/InGaAsP/InP/Pt	GaAs/InGaAsP/InP	1	8.1	0.16	Tandem	2015	37
NiOOH/FeOOH/BVO	DPPOPV	2.2	1	0.036	Tandem	2017	38

H,1%Mo:BiVO/CoP	Two pieces of Si-Ni foam/CoP	5.3	2	0.30-0.32	Tandem	2018	39
W:BiVO/CoPi	a-Si:H//nc-Si:H	5.2	2	0.283	Tandem	2014	40
Mo:BVO/CoO_x-SiC/Pt	FAMAPbI$_3$Br$_3$-Si PV	0.55	2.5	2.59	Tandem	2017	41
Mo:BVO/Fe(Ni)OOH	$MAPbI_3$	6.3	6	0.25	Tandem	2017	42
BVO/TiO_2-PSC	$MAPbI_3$	1.24	6	1	Tandem	2015	43
W:BVO/CoPi	2SHJSi	1.9	7	50	Tandem	2019	44
NiOOH/FeOOH/H,Mo:BVO-Ni_2FeO_x/H,TiO_2/Ti:Fe_2O_3	c-Si	7.7	8	0.24	Tandem	2016	3
NiOOH/FeOOH/Mo:BVO/FTO/Pt/SiO_2	$MAPbI_3$	6.2	10	0.25	Tandem	2016	45
BiVO/$Co(OH)_2$	Carbon-based Lead halide Perovskite	4.6	10	1	Tandem	2019	46
Co–Ci/H,Mo:BVO	$MAPbI_3$/TiO_2-Pt/FTO	4.3	12	0.43	Wired device	2015	47
BVO-F	Polysilicon	5.9	4	5	Parallel	2024	48

NiFe/BVO/SnO_2	Perovskite/Si	7.3	1.39	1	Tandem	2021	49
Au/Ni/ITO/BVO@CoPi	NiO_x/MAPbI_3/PMMA/PCBM/PPDIN_6/Ag	5.3	1.5	0.75	Tandem	2022	50
NiFeOOH/W:BVO	c-Si	2.2	6	100	Parallel	2024	51
NiFeO_x/BVO	c-Si	1.67	40	25	Tandem	2020	52
NiFeO_x/BiVO	c-Si	1.52	40	25	Parallel	2020	52
BVO	Perovskite/Si	7.02	11.25	3.11	Tandem	2023	53
NiFeOOH/Ag/Spiro-OMeTAD/FAPbI_3/TiO_2 /FTO/Cu wire	FAPbI_3 Perovskite PV cell	12.17	14	0.25	Parallel	2024	54
NiFeOOH/Ag/Spiro-OMeTAD/FAPbI_3/TiO_2 /FTO/Cu wire	FAPbI_3 Perovskite PV cell	8.9	4	7.68	Parallel	2024	54
NiFeOOH/Ag/Spiro-OMeTAD/FAPbI_3/TiO_2 /FTO/Cu wire	FAPbI_3 Perovskite PV cell	8.5	4	30.8	Parallel	2024	54
NiFeOOH/Ag/Spiro-OMeTAD/FAPbI_3/TiO_2 /FTO/Cu wire	FAPbI_3 Perovskite PV cell	8.5	4	123.2	Parallel	2024	54

NiFe LDH/Ni/Ni/Perovskite	NiMo/Ni/P erovskite	10.4	1	0.25	Parallel	2023	55
NiFe LDH/Ni/Ni/Perovskite	NiMo/Ni/P erovskite	3.4	1	4	Parallel	2023	55
NiFe LDH/Ni/Perovskite	Perovskite	9.89	24	30	Parallel	2024	56
NiFe LDH/Ni/Perovskite	Perovskite	3.09	7	100	Parallel	2024	56
PTAA/$Cs_xFA_{1-x}Pb(I_{1-y}Br_y)_3$	a/Si:H/c-Si/a/Si:H/Ag	8	72	7.6	Tandem	2024	57
PTAA/$Cs_xFA_{1-x}Pb(I_{1-y}Br_y)_3$	a/Si:H/c-Si/a/Si:H/Ag	6.3	72	362	Tandem	2024	57
Ti doped Fe_2O_3 photoanode	HIT-Si	0.12	13.5	200	Tandem	2020	58
perovskite	Si/Ni foil/NiFe	14	45	1	Tandem	2025	59
Fe_2O_3 photoanode with NiOOH auxiliary electrodes	c-Si	0.68	8.3	200	Tandem	2020	60
WO_3/ZnO	nip a-Si PV cell-SS	3.1		120	Tandem	2006	61
Fe_2O_3	c-Si PV cell-Ti	1.76	8	50	Tandem	2020	60
Gridded WO_3	DSSC	1.41		130.6	Tandem	2011	62

Supplementary Table 10. Relative environmental impacts of natural gas reforming for hydrogen production (normalized to the highest value among three scenarios for each impact category): classified by resources and emissions.

Impact Assessment Categories	Transportation	Electricity	Chemicals	Material	Exhaust Emissions
Climate change	0.86527	0.88745	0.5199	1.1138	96.6
Agricultural land occupation	0.34279	14.95067	7.55704	9.265	0
Fossil depletion	16.22644	15.09219	8.02623	18.15536	0
Freshwater ecotoxicity	0.26525	21.94026	1.99478	0.86989	0
Freshwater eutrophication	0.29795	13.40289	4.47979	3.32852	0
Human toxicity	0.50236	17.63878	4.2028	2.93614	0
Ionising radiation	0.23082	5.53998	2.38233	4.13947	0
Marine ecotoxicity	0.14093	21.82337	2.02467	0.86101	0
Marine eutrophication	3.62968	16.95905	8.52145	6.87522	0
Metal depletion	0.63205	17.76306	3.02791	1.66655	0
Natural land transformation	0.58497	19.29028	8.24526	34.17947	0
Ozone depletion	81.45203	7.61654	4.17773	6.75336	0
Particulate matter formation	3.37288	16.26544	7.76719	8.32359	0
Photochemical oxidant formation	10.7057	17.52352	7.07117	15.46495	0
Terrestrial acidification	3.31392	13.71471	8.31118	7.38081	0
Terrestrial ecotoxicity	0.73902	24.79817	1.10639	1.49856	0
Urban land occupation	0.9906	18.17843	22.44694	26.20281	0
Water depletion	0.25486	19.24246	7.43119	2.84948	0

Supplementary Table 11. Relative environmental impacts of electrocatalytic water splitting for hydrogen production (normalized to the highest value among three scenarios for each impact category): classified by resources and emissions.

Impact Assessment Categories	Transportation	Electricity	Chemicals
Climate change	0.00816	3.4591	2.65273
Agricultural land occupation	0.00327	58.3074	41.69076
Fossil depletion	0.15322	58.85082	40.99596
Freshwater ecotoxicity	0.0025	85.5724	14.42549
Freshwater eutrophication	0.00281	52.21076	47.94359
Human toxicity	0.00474	68.78797	31.2073
Ionising radiation	0.00229	22.70896	77.28903
Marine ecotoxicity	0.00133	85.11509	14.88315
Marine eutrophication	0.03467	66.60774	33.43009
Metal depletion	0.00585	69.25674	30.73741
Natural land transformation	0.00552	74.20331	26.80305
Ozone depletion	0.76835	29.69956	18.01205
Particulate matter formation	0.03187	63.51648	36.45063
Photochemical oxidant formation	0.10106	68.31555	31.55159
Terrestrial acidification	0.03132	53.46279	46.51513
Terrestrial ecotoxicity	0.00697	96.8	3.27564
Urban land occupation	0.00934	70.91458	29.07284
Water depletion	0.0024	75.05494	24.94506

Supplementary Table 12. Relative environmental impacts of photoelectrochemical water splitting for hydrogen production (normalized to the highest value among three scenarios for each impact category): classified by resources and emissions.

Impact Assessment Categories	Transportation	Electricity	Chemicals	Material
Climate change	0.00816	0.49386	0.25837	0.70962
Agricultural land occupation	0.00327	8.33042	4.20572	12.10525
Fossil depletion	0.15322	8.40731	5.92031	11.1334
Freshwater ecotoxicity	0.0025	12.22478	2.80526	0.78992
Freshwater eutrophication	0.00281	7.55366	3.07389	1.75439
Human toxicity	0.04737	9.82684	4.85888	1.85062
Ionising radiation	0.00229	3.24421	1.33561	1.64669
Marine ecotoxicity	0.00133	12.15947	2.82399	0.74677
Marine eutrophication	0.03467	9.41819	3.76299	8.16993
Metal depletion	0.00585	9.89432	4.6933	6.62427
Natural land transformation	0.00552	10.55144	0.86268	6.71529
Ozone depletion	0.76842	4.24318	1.57758	4.07598
Particulate matter formation	0.03187	9.05495	10.94426	11.6044
Photochemical oxidant formation	0.10106	9.76398	7.92716	16.29486
Terrestrial acidification	0.03132	7.64736	7.78484	13.98866
Terrestrial ecotoxicity	0.00993	19.79484	1.22793	0.9673
Urban land occupation	0.00934	10.12706	10.59253	7.48522
Water depletion	0.0024	10.72147	3.44602	2.55763

Supplementary Table 13. Relative environmental impacts of natural gas reforming for hydrogen production (normalized to the highest value among three scenarios for each impact category): classified by unit process.

Impact Assessment Categories	Natural gas extraction and transportation	Steam Conversion	Hydrogen transportation
Climate change	98.6197	1.38029	0.00817
Agricultural land occupation	10.1681	21.9511	0.00327
Fossil depletion	34.9051	22.4377	0.15322
Freshwater ecotoxicity	1.93109	23.1399	0.0025
Freshwater eutrophication	3.98724	17.3844	0.00281
Human toxicity	4.08219	21.1937	0.00474
Ionising radiation	4.80962	8.10851	0.00229
Marine ecotoxicity	1.79447	23.0571	0.00133
Marine eutrophication	11.1111	21.5686	0.03467
Metal depletion	2.94038	20.1389	0.00585
Natural land transformation	36.0465	26.7442	0.00552
Ozone depletion	87.7323	11.4993	0.76842
Particulate matter formation	12.2637	23.4286	0.03187
Photochemical oxidant formation	26.7701	23.8927	0.10106
Terrestrial acidification	11.2047	21.4985	0.03132
Terrestrial ecotoxicity	3.1	25	0.00697
Urban land occupation	27.8651	39.9421	0.00934
Water depletion	3.8341	25.9457	0.0024

Supplementary Table 14. Relative environmental impacts of electrocatalytic water splitting for hydrogen production (normalized to the highest value among three scenarios for each impact category): classified by unit process.

**Impact Assessment Categories**	**Catalyst preparation**	**Hydrogen production**	**Hydrogen collection**	**Hydrogen transportation**
Climate change	0.70118	5.31482	0.09888	0.00817
Agricultural land occupation	11.7443	86.5859	1.66645	0.00327
Fossil depletion	12.0889	86.0762	1.68162	0.15322
Freshwater ecotoxicity	18.2398	79.3127	2.44506	0.0025
Freshwater eutrophication	11.0048	87.4994	1.49305	0.00281
Human toxicity	14.8175	83.2124	1.96541	0.00474
Ionising radiation	5.72378	93.6252	0.64874	0.00229
Marine ecotoxicity	18.5161	79.0505	2.43213	0.00133
Marine eutrophication	14.3791	83.6844	1.90185	0.03467
Metal depletion	30.6267	67.3889	1.97856	0.00585
Natural land transformation	19.7674	78.7168	2.11029	0.00552
Ozone depletion	5.859	41.0083	0.84864	0.76842
Particulate matter formation	22.8571	75.325	1.8022	0.03187
Photochemical oxidant formation	17.8791	80.0376	1.93986	0.10106
Terrestrial acidification	30.4692	26.74	42.7737	0.03132
Terrestrial ecotoxicity	18.1	79.1649	2.8	0.00697
Urban land occupation	17.2475	80.7271	2.02541	0.00934
Water depletion	19.7176	78.1363	2.14616	0.0024

Supplementary Table 15. Relative environmental impacts of photoelectrochemical water splitting for hydrogen production (normalized to the highest value among three scenarios for each impact category): classified by unit process.

Impact Assessment Categories	Catalyst preparation	Hydrogen production	Hydrogen collection	Hydrogen production
Climate change	0.45856	0.90602	0.09888	0.00817
Agricultural land occupation	7.17771	15.7972	1.66645	0.00327
Fossil depletion	8.9051	14.8747	1.68162	0.15322
Freshwater ecotoxicity	9.90663	3.46827	2.44506	0.0025
Freshwater eutrophication	6.37959	4.46571	1.49305	0.00281
Human toxicity	8.15646	6.41458	1.96541	0.00474
Ionising radiation	2.80569	2.77208	0.64874	0.00229
Marine ecotoxicity	9.85464	3.44405	2.43213	0.00133
Marine eutrophication	8.16993	11.2809	1.90185	0.03467
Metal depletion	8.10057	11.1319	1.97856	0.00585
Natural land transformation	9.00763	7.0115	2.11029	0.00552
Ozone depletion	4.00175	5.22376	0.84864	0.76842
Particulate matter formation	7.95604	21.8462	1.8022	0.03187
Photochemical oxidant formation	0.88264	23.1814	1.93986	0.10106
Terrestrial acidification	6.80529	21.0861	1.52947	0.03132
Terrestrial ecotoxicity	15.8074	2.07214	3.98745	0.00993
Urban land occupation	9.33451	13.4608	2.02541	0.00934
Water depletion	9.35615	5.22747	2.14616	0.0024

Supplementary Table 16. Relative environmental impacts of the three scenarios for hydrogen production (normalized to the highest value among three scenarios for each impact category).

Impact Assessment Categories	Scenario 1	Scenario 2	Scenario 3
Climate change	100.00003	6.11999	1.47001
Agricultural land occupation	32.1155	100.00143	24.64466
Fossil depletion	57.50022	100	25.61424
Freshwater ecotoxicity	25.07018	100.00039	15.82246
Freshwater eutrophication	21.50915	100.15716	12.38475
Human toxicity	25.28008	100.00001	16.58371
Ionising radiation	12.2926	100.00028	6.2288
Marine ecotoxicity	24.84998	99.99957	15.73156
Marine eutrophication	35.9854	100.0725	21.38578
Metal depletion	23.08957	100	21.21774
Natural land transformation	62.29998	101.01188	18.13493
Ozone depletion	99.99966	48.47996	10.66516
Particulate matter formation	35.7291	99.99898	31.63548
Photochemical oxidant formation	50.76534	99.9682	34.08706
Terrestrial acidification	32.72062	100.00924	29.45218
Terrestrial ecotoxicity	28.14214	100.08261	22
Urban land occupation	67.81878	99.99676	28.21415
Water depletion	29.77799	100.0024	16.72752

Supplementary References

1 Iandolo, B. & Hellman, A. The role of surface States in the oxygen evolution reaction on hematite. *Angew. Chem. Int. Ed.* **53**, 13404-13408 (2014).

2 Chen, Z. *et al.* Accelerating materials development for photoelectrochemical hydrogen production: Standards for methods, definitions, and reporting protocols. *J. Mater. Res.* **25**, 3-16 (2010).

3 Kim, J. H. *et al.* Hetero-type dual photoanodes for unbiased solar water splitting with extended light harvesting. *Nat. Commun.* **7**, 13380 (2016).

4 Ma, Y., Pendlebury, S. R., Reynal, A., Le Formal, F. & Durrant, J. R. Dynamics of photogenerated holes in undoped $BiVO_4$ photoanodes for solar water oxidation. *Chem. Sci.* **5**, 2964-2973 (2014).

5 Li, Z., Luo, W., Zhang, M., Feng, J. & Zou, Z. Photoelectrochemical cells for solar hydrogen production: current state of promising photoelectrodes, methods to improve their properties, and outlook. *Energy Environ. Sci.* **6**, 347-370 (2013).

6 Zhong, D. K., Choi, S. & Gamelin, D. R. Near-Complete Suppression of Surface Recombination in Solar Photoelectrolysis by "Co-Pi" Catalyst-Modified W:$BiVO_4$. *J. Am. Chem. Soc.* **133**, 18370-18377 (2011).

7 Zhang, X. *et al.* Engineering Single-Atomic Ni-N_4-O Sites on Semiconductor Photoanodes for High-Performance Photoelectrochemical Water Splitting. *J. Am. Chem. Soc.* **143**, 20657-20669 (2021).

8 Kahraman, A., Barzgar Vishlaghi, M., Baylam, I., Sennaroglu, A. & Kaya, S. Roles of Charge Carriers in the Excited State Dynamics of $BiVO_4$ Photoanodes. *J. Phys. Chem. C* **123**, 28576-28583 (2019).

9 Jiang, W. *et al.* Stress-induced $BiVO_4$ photoanode for enhanced photoelectrochemical performance. *Appl. Catal. B* **304**, 121012 (2022).

10 Jian, J. *et al.* Embedding laser generated nanocrystals in $BiVO_4$ photoanode for efficient photoelectrochemical water splitting. *Nat. Commun.* **10**, 2609 (2019).

11 Rahaman, M. *et al.* Solar-driven liquid multi-carbon fuel production using a standalone perovskite–$BiVO_4$ artificial leaf. *Nat. Energy* **8**, 629-638 (2023).

12 Lin, C. *et al.* Photo-Electrochemical Glycerol Conversion over a Mie Scattering Effect Enhanced Porous $BiVO_4$ Photoanode. *Adv. Mater.* **35**, 2209955 (2023).

13 Kim, T. W. & Choi, K. S. Nanoporous $BiVO_4$ photoanodes with dual-layer oxygen evolution catalysts for solar water splitting. *Science* **343**, 990-994 (2014).

14 Meng, Q. *et al.* Efficient $BiVO_4$ photoanodes by postsynthetic treatment: remarkable improvements in photoelectrochemical performance from facile borate modification. *Angew. Chem. Int. Ed.* **58**, 19027-19033 (2019).

15 Xu, L. *et al.* Nitrogen incorporated oxygen vacancy enriched $MnCo_2O_x/BiVO_4$ photoanodes for efficient and stable photoelectrochemical water splitting. *Nano Res.* **17**, 1140-1150 (2024).

16 Ji, Q., Bi, L., Zhang, J., Cao, H. & Zhao, X. S. The role of oxygen vacancies of ABO_3 perovskite oxides in the oxygen reduction reaction. *Energy Environ. Sci.* **13**, 1408-1428 (2020).

17 Pan, J.-B., Wang, B.-H., Shen, S., Chen, L. & Yin, S.-F. Introducing Bidirectional Axial Coordination into $BiVO_4$@Metal Phthalocyanine Core–Shell Photoanodes for Efficient Water Oxidation. *Angew. Chem. Int. Ed.* **62**, e202307246 (2023).

18 Yang, N. *et al.* Insight into the Key Restriction of $BiVO_4$ Photoanodes Prepared by Pyrolysis Method for Scalable Preparation. *Angew. Chem. Int. Ed.* **62**, e202308729 (2023).

19 Wu, H. *et al.* Low-bias photoelectrochemical water splitting via mediating trap states and small polaron hopping. *Nat. Commun.* **13**, 6231 (2022).

20 Zhang, Z., Huang, X., Zhang, B. & Bi, Y. High-performance and stable $BiVO_4$ photoanodes for solar water splitting via phosphorus–oxygen bonded FeNi catalysts. *Energy Environ. Sci.* **15**, 2867-2873 (2022).

21 Song, Y. *et al.* Engineering MoO_x/MXene Hole Transfer Layers for Unexpected Boosting of Photoelectrochemical Water Oxidation. *Angew. Chem. Int. Ed.* **61**, e202200946 (2022).

22 Jian, J. *et al.* Activating a Semiconductor–Liquid Junction via Laser-Derived Dual Interfacial Layers for Boosted Photoelectrochemical Water Splitting. *Adv. Mater.* **34**, 2201140 (2022).

23 Zhang, B. *et al.* Nitrogen-incorporation activates $NiFeO_x$ catalysts for efficiently boosting oxygen evolution activity and stability of $BiVO_4$ photoanodes. *Nat. Commun.* **12**, 6969 (2021).

24 He, B. *et al.* General and Robust Photothermal-Heating-Enabled High-Efficiency Photoelectrochemical Water Splitting. *Adv. Mater.* **33**, 2004406 (2021).

25 Pan, J. B. *et al.* Activity and Stability Boosting of an Oxygen-Vacancy-Rich $BiVO_4$ Photoanode by NiFe-MOFs Thin Layer for Water Oxidation. *Angew. Chem. Int. Ed.* **60**, 1433-1440 (2021).

26 Wang, S. *et al.* In Situ Formation of Oxygen Vacancies Achieving Near-Complete Charge Separation in Planar $BiVO_4$ Photoanodes. *Adv. Mater.* **32**, 2001385 (2020).

27 Jin, B. *et al.* A two-photon tandem black phosphorus quantum dot-sensitized $BiVO_4$ photoanode for solar water splitting. *Energy Environ. Sci.* **15**, 672-679 (2022).

28 Kuang, Y. *et al.* Ultrastable low-bias water splitting photoanodes via photocorrosion inhibition and in situ catalyst regeneration. *Nat. Energy* **2**, 16191 (2016).

29 Lee, D. K. & Choi, K.-S. Enhancing long-term photostability of $BiVO_4$ photoanodes for solar water splitting by tuning electrolyte composition. *Nat. Energy* **3**, 53-60 (2018).

30 Ren, H. *et al.* Manipulation of Charge Transport by Metallic $V_{13}O_{16}$ Decorated on Bismuth Vanadate Photoelectrochemical Catalyst. *Adv. Mater.* **31**, 1807204 (2019).

31 Kim, T. W., Ping, Y., Galli, G. A. & Choi, K.-S. Simultaneous enhancements in photon absorption and charge transport of bismuth vanadate photoanodes for solar water splitting. *Nat. Commun.* **6**, 8769 (2015).

32 Zhang, K. *et al.* Black phosphorene as a hole extraction layer boosting solar water splitting of oxygen evolution catalysts. *Nat. Commun.* **10**, 2001 (2019).

33 Ye, K.-H. *et al.* Enhancing photoelectrochemical water splitting by combining work function tuning and heterojunction engineering. *Nat. Commun.* **10**, 3687 (2019).

34 Chen, Y.-S., Manser, J. S. & Kamat, P. V. All Solution-Processed Lead Halide Perovskite-$BiVO_4$ Tandem Assembly for Photolytic Solar Fuels Production. *J. Am. Chem. Soc.* **137**, 974-981 (2015).

35 Baek, J. H. *et al.* $BiVO_4/WO_3/SnO_2$ Double-Heterojunction Photoanode with Enhanced Charge Separation and Visible-Transparency for Bias-Free Solar Water-Splitting with a Perovskite Solar Cell. *ACS Appl. Mater. Interfaces* **9**, 1479-1487 (2017).

36 Abdi, F. F. *et al.* Efficient solar water splitting by enhanced charge separation in a bismuth vanadate-silicon tandem photoelectrode. *Nat. Commun.* **4**, 2195 (2013).

37 Pihosh, Y. *et al.* Photocatalytic generation of hydrogen by core-shell $WO_3/BiVO_4$ nanorods with ultimate water splitting efficiency. *Sci. Rep.* **5**, 11141 (2015).

38 Peng, Y., Govindaraju, G. V., Lee, D. K., Choi, K.-S. & Andrew, T. L. Integrating a Semitransparent, Fullerene-Free Organic Solar Cell in Tandem with a $BiVO_4$ Photoanode for Unassisted Solar Water Splitting. *ACS Appl. Mater. Interfaces* **9**, 22449-22455 (2017).

39 Kim, J. H., Han, S., Jo, Y. H., Bak, Y. & Lee, J. S. A precious metal-free solar water splitting cell with a bifunctional cobalt phosphide electrocatalyst and doubly promoted bismuth vanadate photoanode. *J. Mater. Chem. A* **6**, 1266-1274 (2018).

40 Han, L. *et al.* Efficient Water-Splitting Device Based on a Bismuth Vanadate Photoanode and Thin-Film Silicon Solar Cells. *ChemSusChem* **7**, 2832-2838 (2014).

41 Iwase, A. *et al.* Solar Water Splitting Utilizing a SiC Photocathode, a $BiVO_4$ Photoanode, and a Perovskite Solar Cell. *ChemSusChem* **10**, 4420-4423 (2017).

42 Xiao, S. *et al.* Integration of inverse nanocone array based bismuth vanadate photoanodes and bandgap-tunable perovskite solar cells for efficient self-powered solar water splitting. *J. Mater. Chem. A* **5**, 19091-19097 (2017).

43 Zhang, X. *et al.* A perovskite solar cell-TiO_2@$BiVO_4$ photoelectrochemical system for direct solar water splitting. *J. Mater. Chem. A* **3**, 21630-21636 (2015).

44 Ahmet, I. Y. *et al.* Demonstration of a 50 cm^2 $BiVO_4$ tandem photoelectrochemical-photovoltaic water splitting device. *Sustainable Energy Fuels* **3**, 2366-2379 (2019).

45 Qiu, Y. *et al.* Efficient solar-driven water splitting by nanocone $BiVO_4$-perovskite tandem cells. *Sci. Adv.* **2**, e1501764 (2016).

46 Li, X. *et al.* $Co(OH)_2/BiVO_4$ photoanode in tandem with a carbon-based perovskite solar cell for solar-driven overall water splitting. *Electrochim. Acta* **330**, 135183 (2020).

47 Kim, J. H. *et al.* Wireless Solar Water Splitting Device with Robust Cobalt-Catalyzed, Dual-Doped $BiVO_4$ Photoanode and Perovskite Solar Cell in Tandem: A Dual Absorber Artificial Leaf. *ACS Nano* **9**, 11820-11829 (2015).

48 Liu, Y., Shang, H., Zhang, B., Yan, D. & Xiang, X. Surface fluorination of $BiVO_4$ for the photoelectrochemical oxidation of glycerol to formic acid. *Nat. Commun.* **15**, 8155 (2024).

49 Yang, J. W. *et al.* Near-complete charge separation in tailored $BiVO_4$-based heterostructure photoanodes toward artificial leaf. *Appl. Catal. B* **293**, 120217 (2021).

50 Tang, S. *et al.* Harvesting of Infrared Part of Sunlight to Enhance Polaron Transport and Solar Water Splitting. *Adv. Funct. Mater.* **32**, 2110284 (2022).

51 Patil Kunturu, P. *et al.* Scaling up $BiVO_4$ Photoanodes on Porous Ti Transport Layers for Solar Hydrogen Production. *ChemSusChem* **17**, e202300969 (2024).

52 Qayum, A. *et al.* An in situ combustion method for scale-up fabrication of $BiVO_4$ photoanodes with enhanced long-term photostability for unassisted solar water splitting. *J. Mater. Chem. A* **8**, 10989-10997 (2020).

53 Xu, Z., Chen, L., Brabec, C. J. & Guo, F. All Printed Photoanode/Photovoltaic Mini-Module for Water Splitting. *Small Methods* **7**, 2300619 (2023).

54 Hansora, D. *et al.* All-perovskite-based unassisted photoelectrochemical water splitting system for efficient, stable and scalable solar hydrogen production. *Nat. Energy* **9**, 272-284 (2024).

55 Choi, H. *et al.* Organometal Halide Perovskite-Based Photoelectrochemical Module Systems for Scalable Unassisted Solar Water Splitting. *Adv. Sci.* **10**, 2303106 (2023).

56 Jeong, W. *et al.* Large-area all-perovskite-based coplanar photoelectrodes for scaled-up solar hydrogen production. *Energy Environ. Sci.* **17**, 3604-3617 (2024).

57 Maragno, A. R. A. *et al.* Thermally integrated photoelectrochemical devices with perovskite/silicon tandem solar cells: a modular approach for scalable direct water splitting. *Sustainable Energy Fuels* **8**, 3726-3739 (2024).

58 Vilanova, A. *et al.* Solar water splitting under natural concentrated sunlight using a 200 cm^2 photoelectrochemical-photovoltaic device. *J. Power Sources* **454**, 227890 (2020).

59 Zhang, D. *et al.* Over 14% unassisted water splitting driven by immersed perovskite/Si tandem photoanode with Ni-based catalysts. *Mater. Today Energy* **48**, 101809 (2025).

60 Landman, A. *et al.* Decoupled Photoelectrochemical Water Splitting System for Centralized Hydrogen Production. *Joule* **4**, 448-471 (2020).

61 Stavrides, A. *et al.* *Use of amorphous silicon tandem junction solar cells for hydrogen production in a photoelectrochemical cell*. Vol. 6340 OP (SPIE, 2006).

62 Lee, W. J., Shinde, P. S., Go, G. H. & Ramasamy, E. Ag grid induced photocurrent enhancement in WO_3 photoanodes and their scale-up performance toward photoelectrochemical H_2 generation. *Int. J. Hydrogen Energy* **36**, 5262-5270 (2011).
