# Supplementary material for: A standalone bismuth vanadate-silicon artificial leaf achieving 8.4% efficiency for hydrogen production
Source: Nat Commun. 2025 Mar 21;16:2792. doi: 10.1038/s41467-025-58102-z (PMC11928484; doi:10.1038/s41467-025-58102-z)
Supplement: Supplementary file 2 — Description of Additional Supplementary Files [file 41467_2025_58102_MOESM2_ESM.pdf]

Description of Additional Supplementary Files

File Name: Supplementary Movie 1
Description: Water splitting of a 3 cm × 3 cm artificial leaf under Xe lamp light illumination. An artificial leaf with dimensions of 3 cm × 3 cm was placed in a photocatalytic activity evaluation system (Beijing China Education Au-Light Co., Ltd., CEL-PAEM-D8). A 1 M potassium borate buffer (pH=9.5) was used as the electrolyte. Before test, the system was vacuumed to remove all gases. A Xe 300 W lamp (CEL-S300, CEAULIGHT) equipped with an AM 1.5G filter was used as the light source. The light intensity at the artificial leaf was carefully calibrated to 100 mW cm^{-2} with the spectrum matching the standard AM 1.5G spectrum.

File Name: Supplementary Movie 2
Description: Water splitting of a 21 cm × 21 cm artificial leaf under natural sunlight illumination. An artificial leaf with dimensions of 21 cm × 21 cm was placed in a home-made quartz reactor with dimensions of 30 cm × 30 cm × 10 cm. A 1 M potassium borate buffer (pH=9.5) was used as the electrolyte. Water splitting was performaned under outdoor sunlight conditions at 14:20 pm on 18th August 2024, at the location of 34.019775°N, 108.92685°E. The intensity of sunlight was measured at 36.0 mW cm^{-2}.
